# Psychosocial Determinants of Sleep Behavior and Healthy Sleep Among Adolescents: A Two-Wave Panel Study

**DOI:** 10.1007/s10964-023-01866-8

**Published:** 2023-09-25

**Authors:** Lea Rahel Delfmann, Maïté Verloigne, Benedicte Deforche, Simon C. Hunter, Greet Cardon, Janneke de Boer, Ann Vandendriessche

**Affiliations:** 1 Department of Public Health and Primary Care, Faculty of Medicine and Health Sciences, Ghent University, Campus UZ-Ghent, Entrance 42, 6th Floor, Corneel Heymanslaan 10, 9000 Ghent, Belgium; 2 Department of Movement and Sports Sciences, Faculty of Medicine and Health Sciences, Ghent University, Watersportlaan 2, 9000 Ghent, Belgium; 3 Movement and Nutrition for Health and Performance Research Group, Faculty of Physical Education and Physical Therapy, Vrije Universiteit Brussel, Pleinlaan 2, 1050 Brussels, Belgium; 4 Department of Psychology, Glasgow Caledonian University, Cowcaddens Road, Glasgow, G4 0BA Scotland UK; 5 Graduate School of Education, University of Western Australia, M428, 35 Stirling Highway, Nedlands WA 6009 Perth, Australia

**Keywords:** Sleep Duration, Sleep Quality, Adolescence, Psychosocial Determinants, Two-Wave Panel Study

## Abstract

To date, it remains unknown which psychosocial determinants identified by several leading behavior change theories are associated with different sleep parameters among adolescents. Therefore, this study investigates whether changes in knowledge about healthy sleep, attitude toward healthy sleep and going to bed on time, self-efficacy to engage in healthy sleep behavior, perceived parental and peer norms, perceived barriers (e.g., worrying, fear of missing out), and perceived support (e.g., bedtime rules, encouragement) related to healthy sleep are associated with changes in adolescents’ sleep duration on school days and free days and sleep quality over a period of 1 year. Two-wave data of 1648 Flemish adolescents (mean age = 15.01, SD = 0.65, 46.3% female) were analyzed using linear models. Increased levels of parental social support, positive attitude towards and perceived advantages of healthy sleep, norm-knowledge, and perceived peer behavior were associated with sleep duration, with parental social support having the strongest association. Increased levels of perceived barriers were associated with decreased levels of sleep quality parameters, and increased levels of self-efficacy, positive attitude, and parental modeling were associated with improved sleep quality parameters, with perceived barriers having the strongest association. The current results indicate that behavior change theories are useful in the context of adolescent sleep behavior and suggest that perceived parental support (i.e., bedtime rules) and perceived barriers are most strongly associated with adolescents’ sleep duration and/or quality.

## Introduction

Poor sleep, characterized by insufficient sleep duration and reduced sleep quality, is an increasing health problem among adolescents (Inchley et al., 2020), and can negatively impact mental health (Jamieson et al., 2020), physical health (Miller et al., 2018), and cognition (Tarokh et al., 2016). It has been suggested that sleep in adolescence is affected by biological factors, contextual factors, as well as psychosocial factors (Becker et al., 2015). Regarding those psychosocial factors or determinants, behavior change theories postulate that knowledge, attitude, self-efficacy, perceived norms, perceived barriers, and perceived social support influence whether humans engage in the behavior or not (Bartholomew Eldredge et al., 2016). However, little research has investigated whether these psychosocial determinants are associated with adolescents’ sleep behavior. This study aims to explore whether changes in knowledge, attitude, self-efficacy, perceived norm, perceived barriers and perceived social support are associated with changes in sleep parameters over a 1-year period using secondary data from Flemish adolescents.

Adolescence is a period in which healthy sleep is particularly important, but might also be particularly disrupted due to a range of different factors, such as a shift in circadian rhythm, detachment from parents, social interests that favor later bedtimes, and increased social media use (Blake et al., 2019). Healthy sleep comprises sleep duration, i.e., the total time spent asleep, and sleep quality. In this study, sleep quality includes the ease of going to bed, the ease of falling asleep and reinitiating sleep, and the ease of returning to wakefulness (Sufrinko et al., 2015). Moreover, daytime sleepiness (Drake et al., 2003) and Sleep Onset Latencies (SOL, Roenneberg et al., 2015) are indicators of sleep quality. A biopsychosocial contextual model of adolescent sleep (Becker et al., 2015) suggests that the biological, contextual, and psychosocial changes one undergoes during adolescence influence (healthy) sleep. Psychosocial factors have previously been suggested to be the most changeable factors, and are therefore most often targeted by public health interventions (Crutzen et al., 2017; Jansen et al., 2015). The Theory of Planned Behavior (TPB, Ajzen, 1991), the Reasoned Action Approach (RAA, Fishbein & Ajzen, 2011), and the Attitude-Social influence-self-Efficacy model (ASE, De Vries et al. (1988)), identify knowledge, attitude, self-efficacy and perceived norms as psychosocial determinants of intending to perform a behavior or not. Actual performance of this behavior may then be hindered by perceived barriers, or facilitated by perceived support (Eldredge et al., 2016). Specifically, knowledge is defined as the understanding that one has of a key concept, attitude is an individual’s positive or negative evaluation of performing a particular behavior, self-efficacy the subjective probability that one is able to perform a behavior, and perceived norms the beliefs about whether one’s environment approves or disapproves from a behavior (Ajzen, 1991).

Previous research suggested that the abovementioned theories are appropriate for understanding, and intervening, in a variety of health behaviors in adolescence including nutrition-related behaviors (Riebl et al., 2015), drinking behavior (Sciglimpaglia et al. (2020)), cyberbullying (Heirman & Walrave, 2012), intimate partner violence (Nardi-Rodríguez et al. (2019)), condom use (Gomes and Nunes (2018)), and safe sex intentions (Armitage & Talibudeen, 2010). Moreover, some developmental specificities such as an increasing need for autonomy, peer norms becoming more important, and increased detachment from parents (Sawyer et al., 2012) might further underscore the importance to investigate psychosocial determinants such as self-efficacy or perceived parental and peer norms regarding healthy sleep during adolescence.

As poor sleep behavior in adolescence is likely to carry over to adult life (Dregan & Armstrong, 2010), it is important to gain insight into the psychosocial determinants that help to explain this behavior already in adolescence. Despite this necessity, little research has investigated the psychosocial determinants identified by behavior change theories in relation to adolescents’ sleep behavior. Previous studies were qualitative (Gruber et al., 2017; Vandendriessche et al. 2022) or only focused on subsets of psychosocial determinants and had a cross-sectional design (Cassoff et al. (2014b); Bonnar et al., 2015; Short et al. (2020)). However, behavior change theories postulate that psychosocial determinants are interrelated, suggesting that they should be investigated together. Qualitative studies indicated that adolescents themselves considered knowledge about sleep, attitude toward sleep and going to bed on time, self-efficacy regarding going to bed on time, perceived parental and peer norms, and perceived barriers and support to going to bed on time to be important factors influencing their sleep behavior. These studies, however, advocated for the use of quantitative methods in large samples to confirm their findings (Gruber et al., 2017; Vandendriessche et al., 2022). Studies using quantitative methods, but only focusing on a subset of psychosocial determinants, found that attitude and parental social support might be important psychosocial determinants of sleep (Cassoff et al. (2014b); Bonnar et al., 2015; Short et al. (2020)). Another study indicated that emotional and behavioral difficulties were longitudinally related to increased sleep problems in adolescents (Kortesoja et al., 2020). Although this study did not specifically focus on the psychosocial determinants identified by behavior change theories, experiencing emotional and behavioral difficulties might be seen as an important perceived barrier toward healthy sleep.

## Current Study

As outlined above, it has not yet been investigated whether the psychosocial determinants knowledge, attitude, self-efficacy, perceived norms, perceived barriers, and perceived social support are useful to explain sleep behavior in adolescents. The current study aims to explore whether changes in psychosocial determinants associate with changes in several sleep parameters over a 1-year period, using data from Flemish adolescents. Based on abovementioned theories, all psychosocial determinants are expected to be associated with sleep parameters. However, based on previous research, it is specifically hypothesized that increases in perceived social support and positive attitudes toward sleep are more strongly associated with improved sleep outcomes. Moreover, it is hypothesized that a decrease in perceived barriers (e.g., emotional difficulties) is associated with improved sleep parameters, as previous research has shown that psychosocial wellbeing is important for good sleep in adolescence.

## Methods

Data from a cluster-randomized controlled trial were used, in which an intervention to promote healthy sleep in adolescents was developed, implemented and evaluated using a co-creation approach (Vandendriessche et al., 2020). Co-creation entailed active involvement of the target group in the development of the intervention. Sleep parameters and psychosocial determinants were assessed by administering questionnaires at four time points: T0 (before the co-creation process), T1 (after the co-creation process and before intervention start), T2 (directly after the intervention), and T3 (6 months after the end of the intervention). For the present study, data from T0 (November 2017) and T1 (November 2018) were analyzed. As adolescents had not received any kind of intervention between those time points, natural changes in determinants and sleep over a 1-year period could be observed in both intervention- and control group participants. Fourteen adolescents took part in the co-creation process to develop the intervention and were therefore excluded from analysis, resulting in a sample of 1648 adolescents.

Six schools in Flanders (Belgium) were recruited via phone calls, during which the study was explained to them. All eight-and ninth-graders from these schools were invited to participate in the original study. Parents were asked to give consent 1 week before start of data collection. Students were excluded from the study if a sleeping problem had been previously diagnosed. 1648 students (mean age = 15.19, SD = 0.69, 44% girls) from 6 schools (*n* = 640, *n* = 420, *n* = 261, *n* = 70, *n* = 152, *n* = 105) were included in the analyses. 77% of the students were born in Belgium, and 65% had parents with a high educational level. Between T0 (November 2017) and T1 (November 2018), 370 students dropped out of the study. Drop-out analyses were performed to investigate whether students who dropped out differed characteristically from students who did not. Participant characteristics and descriptives are shown in Tables 1 and 2.

**Table 1:** Descriptive statistics of the sample

Baseline characteristic	*n*	%
Biological sex		
Girls	730	44
Boys	918	56
Place of Birth		
Born within Belgium	1272	77
Born in Europe	90	5
Born outside Europe	154	9
SES^a^		
Low educational level	171	14
Average educational level	259	21
High educational level	798	65

^a^SES calculated based on parental educational level (low/average/high)

**Table 2:** Sleep variables at different measurement points

Sleep variable	T0			T1			Δ			*F(DF)*
	*M*	SD	*N*	*M*	SD	*N*	*M*	SD	*N*	
Sleep duration schooldays (hours)	8.01	0.98	1275	7.77	0.95	1232	−0.25	0.88	954	57.84(737, 6)^***^
Sleep duration free days (hours)	9.63	1.4	1264	9.41	1.33	1223	−0.25	1.32	944	48.68(721, 6)^***^
General sleep quality^a^	34.37	4.98	1292	35.14	4.4	1254	0.54	4.48	984	50.75(759, 6)^***^
Daytime sleepiness^b^	22.53	5.22	1295	22.4	5.11	1231	−0.02	4.5	974	86.51(752, 6)^***^
Sleep Onset Latency schooldays (mean)^c^	0.44	0.4	1325	0.37	0.35	1273	−0.06	0.36	1022	61.17(787, 6)^***^
Sleep Onset Latency free days (mean)^c^	0.34	0.36	1321	0.3	0.32	1270	−0.03	0.36	1016	28.61(781, 6)^***^
Knowledge^d^	2.66	0.40	1315	2.94	0.30	1266	0.29	0.49	1010	4.99(784, 6)^***^
Norm knowledge^e^	68%	0.47	1317	71%	0.46	1256	0%	0.58	1005	5.16(779, 6)^***^
Attitudes^f^	3.74	0.85	1315	3.80	0.81	1254	0.03	0.83	1000	38.35(777, 6)^***^
Perceived advantages^f^	3.24	0.91	1299	3.47	0.74	1236	0.21	0.99	982	13.09(761, 6)^***^
Self-efficacy^f^	3.19	0.83	1262	3.29	0.72	1215	0.05	0.85	953	22.58(736, 6)^***^
Modelling peers^f^	2.78	0.82	1207	2.72	0.75	1220	−0.05	0.88	908	23.20(704, 6)^***^
Perceived norm peers^f^	3.1	0.83	1143	3.01	0.75	1213	−0.10	0.97	860	7.26(658, 6)^***^
Modelling parents^f^	3.4	0.87	1287	3.38	0.78	1226	−0.03	0.80	972	38.17(752, 6)^***^
Perceived norm parents^f^	3.78	0.76	1284	3.77	0.73	1224	−0.01	0.80	966	26.15(46, 6)^***^
Perceived norm parents, related to adolescent behavior^f^	4.41	0.83	1256	4.34	0.82	1211	−0.06	0.91	949	19.30(733, 6)^***^
Perceived barriers^g^	2.48	0.63	1312	2.48	0.59	1248	0.03	0.63	998	36.31(776, 6)^***^
Perceived parental support (encouragement)^g^	4.09	0.96	1256	3.93	1.04	1211	−0.11	1.10	949	19.89(732, 6)^***^
Perceived parental support (bedtime rules school days)^h^	3.36	1.28	1322	3.04	1.38	1272	−0.29	1.24	1020	58.28(785, 6)^***^
Perceived parental support (bedtime rules free days)^h^	2.14	1.13	1320	2	1.08	1270	−0.14	1.04	1018	51.22(84, 6)^***^

***p < 0.001

^a^s-ASWS: Range 10–50 with higher scores indicating better sleep quality

^b^PDSS: Range 8–40 with higher scores indicating more daytime sleepiness

^c^Sleep onset latencies (mean hours)

^d^Answer possibilities ranging from *1* *=* *definitely true* to *4* *=* *definitely wrong*

^e^Open answer, recoded into 0 = wrong and 1 = right. Percentage of right answers is reported in the table

^f^5-point scale, higher scores reflecting a more positive relation to healthy sleep

^g^5-point scale, higher scores indicating more perceived barriers toward healthy sleep

^h^5-point scale, higher scores indicating more frequent bedtime rules

### Measurements

All measurements consisted of questionnaires and were administered online using SurveyMonkey (SurveyMonkey, 2019) in two schools at T0 and one school at T1, or on paper and processed using TeleForm (Simac, 2019) in four schools at T0 and five schools at T1. Participants filled out the questionnaires during class hours under supervision of a researcher (AV) or a teacher.

#### Sleep parameters

Sleep parameters assessed in the current study included sleep duration on school days and free days, and parameters for sleep quality (general sleep quality, daytime sleepiness, and Sleep Onset Latencies (SOL) on school days and free days). Items from several validated questionnaires were translated into Flemish, and the translation was checked by two researchers (MV and BD). Translated items were then tested for comprehensiveness in five adolescents (13–16 years) using a think-aloud method.

##### Sleep duration

Sleep duration on school days and on free days was assessed using items from the Munich Chronotype Questionnaire (MCTQ; Roenneberg et al., 2015; Cheung et al., 2022) for children. The items (1) *“When do you usually go to bed?”*, (2) *“When do you usually try to fall asleep?”*, (3) *“How long does it usually take before you fall asleep, from the moment you try to fall asleep until the moment that you really sleep?”* and (4) *“When do you usually wake up?”* were used to calculate sleep duration (Vandendriessche et al., 2021). Response options were intervals of 15 minutes, which were then recoded using the midpoint method. For example, the answer option 21:01 to 21:15 was recoded to 21:08. Sleep onset was calculated by adding the time of trying to go to sleep (item 2) and the time it really took to fall asleep (item 3). Then, sleep duration was computed by calculating the difference between sleep onset and the time of waking up (item 4). The MCTQ showed acceptable validity in adolescents as compared to the Composite Scale of Morningness (*r* = −0.62; Randler, 2008).

##### Sleep onset latencies (SOL)

SOLs on school days and free days were assessed using item 3 *(“How long does it usually take before you fall asleep, from the moment you try to fall asleep until the moment that you really sleep?”)* of the MCTQ as an indicator for sleep quality.

##### General sleep quality

Sleep quality in the previous month was assessed using the short Adolescent Sleep Wake Scale (s-ASWS; Essner et al., 2015). This scale assesses falling asleep and reinitiating sleep, returning to wakefulness, and going to bed. An example item is *“When I wake up during the night, I find it difficult to go back to sleep”*. Answer possibilities ranged from 1 = *“never”* to 5 = *“always”*. Reversed items were recoded and a sum score was calculated with higher scores indicating higher sleep quality. The s-ASWS has been shown to have good internal consistency (α = 0.74–0.84) in healthy samples (Sufrinko et al., 2015), and α_T0_ = 0.74 and α_T1_ = 0.71 in the current sample.

##### Daytime sleepiness

Daytime sleepiness in the previous month was assessed using the Pediatric Daytime Sleepiness Scale (PDSS; Drake et al., 2003). The PDSS consists of eight items. An example item is *“How often do you feel sleepy during class?”*. Answer possibilities ranged from 1 = *“never”* to 5 = *“always”*. Reversed items were recoded, and a sum score was calculated, with higher scores reflecting higher daytime sleepiness. The PDSS has been shown to have a significant linear relation with total sleep time and good internal consistency (α_T0_ = 0.74; α_T1_ = 0.72).

#### Psychosocial determinants of sleep behavior

The questionnaire to assess psychosocial determinants of adolescents’ sleep behavior was developed based on the results of a focus group study about psychosocial determinants of sleep (Vandendriessche et al., 2022). Using a think-aloud method, the questionnaire was first tested for comprehensiveness in five adolescents (aged 13–16). Further, test-retest reliability ranged from −0.04 to 0.86 in a sample of 34 adolescents (aged 13–16). Items with ICC < 0.40 were adapted or dropped. Lastly, construct validity was tested in 22 adolescents (aged 13–16) by having three researchers (AV, IVE, and EV) conduct cognitive interviews about the same topics as the questionnaire. After the interview, another researcher (AV, IVE, or EV) filled in the questionnaire based on the adolescents’ answers during the interview. These answers were then compared to the answers given by the adolescents themselves on the identical questionnaire. Percentage of agreement ranged from 4.6% to 95.2%. Items with percentage of agreement <40% were adapted or dropped.

Knowledge was assessed using 13 statements around general sleep knowledge, sleep hygiene, and impact of sleep on health. An example item is *“Drinking alcohol (even in small amounts) helps you to sleep well”*. Questions could be answered on a four-point scale, ranging from 1 = *“definitely true”* to 4 = *“definitely not true”*. Right answers were recoded, with a higher score representing more knowledge. Knowledge about the recommended norm of sleeping 8 h per night was assessed with an open question *(“How many hours of sleep does someone of your age group need, according to you?”)*. This item was recoded into 1 = *“right”* and 0 = *“wrong”* answers. Internal consistency for the knowledge subscale was minimally acceptable (*α*_*T0*_ = *0.67*, α_T1_ = *0.67)*. Attitude toward sleep duration and quality, as well as going to bed on time was assessed using two items (for example *“I find it important to sleep sufficient and sufficiently well”*), which could be answered on a five-point scale, ranging from 1 = “*completely disagree”* to 5 = *“completely agree”*. Relatedly, perceived advantages of going to bed on time were assessed with seven items (for example *“Going to bed on time has the advantage that I am in a good mood the next day”*), which could be answered on a five-point scale ranging from 1 = *“completely disagree”* to 5 = *“completely agree”*. Subscales for attitude and perceived advantages showed acceptable internal consistency (*α*_*T0*_ = *0.71, α*_*T1*_ = *0.78* and *α*_*T0*_ = *0.81, α*_*T1*_ = *0.82*, respectively*)*. Self-efficacy toward going to bed on time was assessed using eight items (for example *“I think that I can go to bed on time even if I miss a specific TV program”)*, which could be answered on a five-point scale ranging from 1 = “completely disagree” to 5 = “completely agree”. The self-efficacy subscale showed acceptable internal consistency (*α*_*T0*_ = *0.80, α*_*T1*_ = *0.74)*. Perceived norms regarding sleep duration, sleep quality, and going to bed on time were assessed using three items addressing perceived parental norms and three items assessing perceived peer norms (for example *“My peers think that watching specific TV programs is more important than getting enough sleep.”*). Moreover, four items assessed parental modeling and four items assessed peer modeling (for example *“My best friends go to bed on time.”*). Items assessing perceived norms could be answered on a five-point scale ranging from 1 = *“completely disagree”* to 5 = *“completely agree”*. Items assessing modeling could be answered on a five-point scale ranging from 1 = *“never”* to 5 = *“always”*, while *“I don’t know”* could be indicated as an answer as well. Internal consistency of the perceived norm subscales were minimally acceptable for parents and peers (*α*_*T0*_ = *0.71, α*_*T1*_ = *0.66* and *α*_*T0*_ = *0.65, α*_*T1*_ = *0.65*, respectively*)*. Perceived barriers toward going to bed on time was assessed using eleven items. An example item is *“What prevents me from going to bed on time is that I have too much stress caused by my school work”*. Items could be answered on a five-point scale ranging from 1 = “*completely disagree”* to 5 = *“completely agree”*. Internal consistency was minimally acceptable (*α*_*T0*_ = *0.67, α*_*T1*_ = *0.69)*. Perceived parental support was assessed with two items regarding bedtime rules on school days and free days. An example item is “*On school days, I have a set bedtime, imposed by my parents*”. Items could be answered on a five-point scale ranging from 1 = *“never”* to 5 = *“always”*. Moreover, one item assessed perceived parental encouragement regarding healthy sleep behavior *(“My parents encourage me to go to bed on time”*), and could be answered on a five-point scale ranging from 1 = *“completely disagree”* to 5 = *“completely agree”*. Internal consistency was minimally acceptable *(α*_*T0*_ = *0.62, α*_*T1*_ = *0.69)*. An overview of all items including Cronbach’s alphas is provided in Appendix 1.

### Data Analysis

The current study explored whether changes in psychosocial determinants of adolescent sleep behavior from T0 to T1 were associated with changes in sleep parameters from T0 to T1. This approach considers that psychosocial determinants are most likely not static over time. All analyses were carried out using the statistical package R, version 4.3.1 (R Core Team, 2023). Alpha levels of 0.05 were used for all statistical tests.

There were 23.43% of missing values among the primary study variables (22.13% at T0 and 24.74% at T1), meaning that only 76.57% of the 1648 participants in the sample would have been available for analysis under traditional listwise deletion analysis methods. Especially for calculating the difference scores (T1-T0) this would have posed a problem. Consequently, Multiple Imputation by Chained Equations (MICE; Van Buuren & Groothuis-Oudshoorn, 2011) was used to impute missing data. MICE iteratively fills in missing data for each variable in the dataset by using data from other variables in the dataset until convergence is met. Ten imputations were generated using the predictive mean matching method, as well as passive imputation to derive the transformed difference scores of the outcome and predictor variables during the imputation algorithm. All variables used in the linear models (see below) were included as predictor variables in the predictor matrix. Difference scores were used to predict outcome variables, whereas raw data were used to predict difference scores. Analyses run on the ten separate datasets were pooled (Rubin, 1987). Sensitivity analyses were run with non-imputed data.

To check if assumptions for linear regression analysis were met, distributions of the outcome variables were visually checked using histograms. Linearity was checked using residual vs. fitted plots and multicollinearity was checked with the variance inflation factor. As ICCs were close to zero, random effects were not added and we opted for more parsimonious linear models. However, sensitivity analyses were run with mixed linear models for models if models did not suffer from singularity. In total, six linear models were fitted, with the difference scores (T1-T0) of the respective sleep parameters (sleep duration on school days (1), and on free days (2), general sleep quality (3), daytime sleepiness (4), SOL on school days (5) and on free days (6)) as outcome variables, and the difference scores of psychosocial determinants (T1-T0) as predictor variables. Baseline values of sleep parameters and psychosocial determinants were included as covariates to control for baseline differences among participants, and predictor variables were allowed to covary with one another.

As an additional approach to establish determinant relevance, Confidence Interval-Based Estimation of Relevance (CIBER: Crutzen et al., 2017) was used. CIBER assesses the univariate distributions of each determinant based on means, and allows visual inspection of the room for improvement of each determinant, as well as its associations with the behavioral outcomes based on correlations. As CIBER is a visual approach to data analysis and does not allow to test for significance, this will not be described in further detail in this article. However, results are provided in Appendix 2.

## Results

The following variables were found to be related to drop-out: biological sex, SES as assessed with parental educational level, sleep duration on school days, sleep quality, and attitude toward sleep. Boys, participants with lower educated parents, lower sleep duration on school days, lower sleep quality, and less positive attitude toward sleep had higher chances of dropping out at T1. No statistically significant differences were observed for age, place of birth, sleep duration on school days, and daytime sleepiness. Associations between the different sleep parameters were investigated as well, with correlations ranging from −0.05 for SOL on school days at T0 and sleep duration on free days at T1, to 0.67 for SOL on school days at T1 and SOL on free days at T1. Moreover, associations between psychosocial determinants ranged from <0.001 for norm knowledge at T1 and perceived barriers at T0, to 0.58 for bedtime rules on school days at T1 and bedtime rules on school days at T0. Lastly, associations between sleep parameters and psychosocial determinants ranged from <0.001 for norm knowledge at T1 and sleep duration on free days at T0, to 0.31 for general sleep quality at T1 and self-efficacy at T1. Correlation tables can be found in Appendix 3.

Associations between changes in psychosocial determinants and changes in sleep duration and quality, including total variance explained (R^2^) are provided in Tables 3–5. Unstandardized B coefficients show the increase of sleep duration and sleep quality for every one-unit increase of the determinant. Standardized beta coefficients show the relative strength of the association of each psychosocial determinant to the sleep parameters. CIBER results (Appendix 2) were mostly in line with results from regression analyses.

**Table 3:** Results of linear models: Associations of changes in psychosocial determinants with changes in sleep duration

	Sleep Duration									
	Sleep duration on schooldays					Sleep duration on free days				
Explained variance R²	33.44%					28.11%				
Determinant	B	*ß*	95% C.I.		*p*	B	*ß*	95% C.I.		*p*
			LL	UL				LL	UL	
Knowledge	−0.10	0.00	−0.29	0.08	0.30	−0.01	0.03	−0.30	0.27	0.93
Norm-knowledge	0.13	0.05	0.00	0.25	0.04	−0.08	0.04	−0.27	0.10	0.37
Attitudes	0.20	0.07	0.11	0.29	<0.001	0.06	0.02	−0.08	0.19	0.41
Perceived advantages	0.08	0.04	0.00	0.17	0.06	0.22	0.04	0.08	0.35	0.001
Self-efficacy	0.05	0.01	−0.04	0.14	0.28	0.04	0.01	−0.10	0.18	0.55
Modelling peers	0.10	0.05	0.02	0.18	0.01	−0.00	0.01	−0.12	0.12	0.97
Perceived norm peers	−0.08	−0.04	−0.15	0.01	0.05	0.05	0.00	−0.07	0.17	0.40
Modelling parents	−0.004	0.01	−0.09	0.08	0.92	0.04	0.01	−0.08	0.17	0.50
Perceived norm parents	−0.04	−0.02	−0.13	0.05	0.39	0.04	0.03	−0.09	0.18	0.54
Perceived norm parents, related to adolescent behavior	−0.04	0.01	−0.13	0.04	0.32	0.04	0.00	−0.08	0.17	0.50
Perceived barriers	−0.07	−0.04	−0.18	0.05	0.25	−0.08	−0.04	−0.25	0.09	0.37
Perceived parental support (encouragement)	−0.03	−0.00	−0.10	0.04	0.37	−0.04	0.02	−0.14	0.05	0.38
Perceived parental support (bedtime rules school days)	0.14	0.09	0.09	0.18	<0.001	–	–	–	–	–
Perceived parental support (bedtime rules free days)	–	–	–	–	–	0.23	0.08	0.13	0.32	<0.001

**Table 4:** Results of linear models: Associations of changes in psychosocial determinants with changes in sleep quality (general sleep quality and daytime sleepiness)

	General sleep quality					Daytime sleepiness				
Explained variance R²	47.65%					29.35%				
Determinant	B	*ß*	95% C.I.		*p*	B	*ß*	95% C.I.		*p*
			LL	UL				LL	UL	
Knowledge	−0.28	−0.01	−1.14	0.57	0.52	0.75	0.04	−0.24	1.75	0.14
Norm-knowledge	−0.29	−0.01	−0.84	0.26	0.30	−0.57	−0.01	−1.20	0.06	0.07
Attitudes	0.47	0.06	0.07	0.87	0.02	0.44	−0.01	−0.02	0.90	0.06.13
Perceived advantages	0.31	0.06	−0.08	0.71	0.12	0.08	−0.01	−0.37	0.52	0.74
Self-efficacy	0.66	0.06	0.25	1.07	0.001	−0.78	−0.04	−1.25	−0.31	0.001
Modelling peers	0.09	0.02	−0.26	0.46	0.59	−0.37	−0.04	−0.79	0.05	0.08
Perceived norm peers	−0.14	0.02	−0.50	0.21	0.44	0.12	0.00	−0.29	0.54	0.55
Modelling parents	0.28	0.05	−0.10	0.66	0.15	−0.53	−0.02	−0.97	0.09	0.02
Perceived norm parents	−0.14	0.00	−0.50	0.22	0.44	0.20	−0.02	−0.26	0.68	0.38
Perceived norm parents, related to adolescent behavior	0.24	0.00	−0.15	0.63	0.22	−0.37	−0.03	−0.81	0.08	0.11
Perceived barriers	−2.62	−0.18	−3.14	−2.11	<0.001	1.47	0.10	0.90	2.06	<0.001
Perceived parental support (encouragement)	−0.13	−0.01	−0.44	0.17	0.38	−0.07	0.01	−0.42	0.29	0.70
Perceived parental support (bedtime rules school days)	−0.13	−0.02	−0.39	0.11	0.38	−0.06	0.00	−0.34	0.23	0.69
Perceived parental support (bedtime rules free days)	−0.14	−0.01	−0.44	0.16	0.26	−0.06	−0.02	−0.40	0.28	0.69

**Table 5:** Results of linear models: Associations of changes in psychosocial determinants with changes in sleep quality (sleep onset latencies (SOL) on school days and free days)

	SOL school days					SOL free days				
Explained variance *R*²	33.89%					34.72%				
Determinant	B	*ß*	95% C.I.		*p*	B	*ß*	95% C.I.		*p*
			LL	UL				LL	UL	
Knowledge	0.04	0.05	−0.03	0.11	0.26	0.03	0.03	−0.05	0.11	0.45
Norm-knowledge	−0.02	−0.03	−0.07	0.02	0.29	−0.00	−0.02	−0.05	0.04	0.86
Attitudes	−0.01	−0.00	−0.05	0.02	0.49	−0.01	−0.01	−0.04	0.03	0.64
Perceived advantages	0.01	−0.02	−0.02	0.04	0.61	0.01	0.00	−0.02	0.05	0.54
Self-efficacy	−0.01	−0.02	−0.04	0.03	0.77	0.03	0.01	−0.01	0.06	0.16
Modelling peers	−0.01	0.01	−0.04	0.02	0.46	−0.01	0.01	−0.05	0.02	0.39
Perceived norm peers	0.00	0.01	−0.03	0.03	0.91	−0.01	−0.01	−0.04	0.02	0.51
Modelling parents	−0.01	0.01	−0.04	0.02	0.58	−0.02	−0.02	−0.05	0.02	0.37
Perceived norm parents	0.03	0.03	−0.01	0.06	0.14	0.03	0.03	0.00	0.07	0.07
Perceived norm parents, related to adolescent behavior	0.01	0.00	−0.02	0.05	0.43	0.00	−0.01	−0.03	0.03	0.98
Perceived barriers	0.11	0.09	0.07	0.15	<0.001	0.08	0.08	0.04	0.13	<0.001
Perceived parental support (encouragement)	−0.00	−0.00	−0.03	0.02	0.68	−0.01	−0.00	−0.04	0.01	0.24
Perceived parental support (bedtime rules school days)	−0.00	0.00	−0.02	0.02	0.86	–	–	–	–	–
Perceived parental support (bedtime rules free days)	–	–	–	–	–	0.01	0.02	−0.01	0.03	0.40

### Associations of Change in Psychosocial Determinants with Change in Sleep Duration

Bedtime rules (i.e., perceived parental support), attitude toward healthy sleep, perceived advantages of healthy sleep, knowledge of the norm to sleep at least 8 h per night, and perceived peer behavior were found to be significantly associated with sleep duration on school days and/or on free days. Specifically, a one-unit increase in bedtime rules was associated with an increase in sleep duration of 8 min and 4 s on school days and 13 min and 8 s on free days (*B* = 0.14, *β* = 0.09, *p* < 0.001; *B* = 0.23, *β* = 0.08, *p* < 0.001). Moreover, a one-unit increase in positive attitude toward healthy sleep was associated with an increase in sleep duration of 12 min on school days (*B* = 0.20, *β* = 0.07, *p* < 0.001). Relatedly, a one-unit increase in the perceived advantages of healthy sleep was significantly associated with an increase in sleep duration of 13 min and 2 s on free days (*B* = 0.22, *β* = 0.04, *p* = 0.001). A one-unit increase in knowledge of the norm of sleeping 8 h per night was associated with an increase in sleep duration of 7 min and 8 s (*B* = 0.13, *β* = 0.05, *p* = 0.04) on school days. Lastly, a one-unit increase in modeling by peers was associated with increases in sleep duration of 6 min on school days (*B* = 0.10, *β* = 0.05, *p* = 0.01), while a one-unit increase in peer norms was associated with a decrease of 4 min and 8 s at school days (*B* = −0.08, *β* = −0.04, *p* = 0.05). Changes in the remaining psychosocial determinants were not significantly associated with changes in sleep duration.

### Associations of Change in Psychological Determinants with Change in Sleep Quality

Perceived barriers toward going to bed on time, attitude toward healthy sleep, self-efficacy toward engaging in healthy sleep behavior, and parental modeling were found to be significantly associated with sleep quality parameters. Specifically, a one-unit increase in perceived barriers was associated with a decrease of 2.62 on a scale of 60 for general sleep quality (*B* = −2.62, *β* = −0.18, *p* < 0.001) and an increase of 1.47 on a scale of 40 for daytime sleepiness (*B* = 1.47, *β* = 0.10, *p* < 0.001). Further, a one-unit increase in perceived barriers was associated with an increase in Sleep Onset Latencies of 6 min and 6 s on school days and 4 min and 8 s on free days (*B* = 0.11, *β* = 0.09, *p* < 0.001; *B* = 0.09, *β* = 0.08, *p* < 0.001). One-unit increases in self-efficacy were associated with an increase of 0.66 on a scale of 60 for general sleep quality (*B* = 0.66, *β* = 0.06, *p* = 0.001), and a decrease of 0.78 on a scale of 40 for daytime sleepiness (*B* = −0.78, *β* = −0.04, *p* = 0.001). Further, one-unit increases in positive attitude toward healthy sleep were associated with increases of 0.47 on a scale of 60 for general sleep quality (*B* = 0.47, *β* = 0.06, *p* = 0.02). One-unit increases in parental modeling were associated with a decrease of 0.53 on a scale of 40 for daytime sleepiness (*B* = −0.53, *β* = −0.02, *p* = 0.02). Changes in the remaining psychosocial determinants were not significantly associated with changes in sleep quality.

### Sensitivity Analyses

Sensitivity analyses were run for all models without imputation, Moreover, mixed linear models were included as sensitivity analyses for those models which did not suffer from singularity. Results were similar to original results (see Appendix 4).

## Discussion

While deepening knowledge on the most important changeable factors of adolescent sleep is important to understand adolescent sleep and eventually develop healthy sleep interventions, little research has investigated the psychosocial determinants identified by leading behavior change theories in relation to adolescent sleep. The current study explored whether changes in psychosocial determinants (i.e., knowledge, attitudes, perceived norms, self-efficacy, perceived barriers, and perceived social support), were associated with changes in adolescent sleep duration and sleep quality parameters. Changes in perceived parental social support (i.e., having bedtime rules), positive attitudes toward and perceived advantages of healthy sleep, perceived peer behavior, and norm knowledge were associated with changes in sleep duration, with parental social support and attitude having the strongest association. Changes in perceived barriers, self-efficacy, attitude, and perceived parental behavior were associated with changes in sleep quality parameters, with perceived barriers having the strongest association. The current results confirm the hypothesis that perceived social support, attitude, and perceived barriers are important psychosocial factors related to adolescent sleep and are in line with previous research (Cassoff et al. (2014b); Bonnar et al. (2015); Short et al. (2020); Kortesoja et al., (2020)). However, as is suggested by leading behavior change theories, other psychosocial determinants also seem to play a role.

Bedtime rules (i.e., perceived support from parents), had the strongest association with sleep duration, with increases of 8.4 min on weekdays and 13.8 min on weekends. This indicates that the structure a family can offer is important for adolescents’ sleep health, despite their increased need for independence and autonomy, including detachment from parents (Spear & Kulbok, 2004). While effect sizes are small, this finding is still promising, considering that increases of 15 min in sleep duration have been found to be clinically relevant (Perfect et al., 2016). These results are in line with previous research, in which structured home environments and family support were found to improve healthy sleep in children and adolescents (Bally & van Grieken (2020); Leonard & Khurana, 2022). Interestingly, encouragement of healthy sleep behavior was not found to be associated with sleep parameters, indicating that tangible bedtime rules might have a greater impact on adolescents’ sleep than simple encouragement. A significant association between increased levels of parental modeling and decreased levels of daytime sleepiness suggests that parents might be important figures when it comes to aspects of adolescent sleep quality, as well. While the current findings underscore the importance of parents for adolescent healthy sleep, it is unclear how this applies to adolescents who are not supported by their parents or do not have a parent or guardian figure in their lives. In that sense, the current findings might not be generalizable to a less privileged sample.

While parental influence might gradually decrease during adolescence, more time is spent with peers and the norms of peer networks become increasingly important (Ryan, 2000; Wang et al., 2018). This is also reflected in the current results, as positive peer modeling and perceived peer sleep norms were found to be significantly associated with sleep duration on school days. Nowadays, social media use allows adolescents to closely monitor when peers are online, until when they send messages, and consequently when they go to bed, which might further explain these results. Moreover, previous studies indicated that adolescents were embarrassed to tell peers they would prefer sleeping to chatting (Vandendriessche et al., 2022), underscoring the importance of peer norms in relation to bedtimes. Sleep quality was, however, not associated with perceived peer behavior. Due to the reasons stated above, sleep duration and bedtimes might be more salient or observable to peers than how well adolescents sleep, e.g., how long it takes them to fall asleep, and how many times they wake up during the night.

Increased levels of positive attitudes and relatedly perceived advantages of healthy sleep behavior were found to be associated with improved levels of sleep duration on school days and free days, as well as with general sleep quality. While attitudes were less strongly associated with sleep duration than bedtime rules, the increase of 12 min is quite mentionable. These results confirm the hypothesis that attitudes are important psychosocial determinants of adolescent sleep. Moreover, this is in line with previous research showing that attitudes were strong predictors for other health behaviors in adolescence, namely nutrition behavior (Riebl et al., 2015) and cyberbullying (Heirman & Walrave, 2012). The current findings suggest the need to improve adolescents’ positive attitudes toward healthy sleep and going to bed on time to improve healthy sleep. Considering that adolescents have been shown to prioritize other activities over sleep (Vandendriessche et al.,^ 2022^), this might be challenging. However, behavior change techniques like the direct experience of rewarding outcomes (Maibach & Cotton, 1995), (e.g., by keeping a sleep-mood diary) or repeated exposure to a stimulus (Zajonc, 2001), (e.g., by using prompts in a mobile application) have been shown to bring about attitude change. Interestingly, an increased level of knowledge about sleep, which has been proposed to be the first step toward developing a positive attitude (Cain et al., 2011), was not found to be associated with improved levels of sleep parameters, except for norm knowledge and sleep duration on school days. This is in line with previous studies showing that knowledge about short-term negative consequences of poor sleep did not translate into healthier sleep behavior among adolescents (Cassoff et al., 2014a; Gruber et al., 2017). Moreover, a cross-sectional study in college students found attitude, but not knowledge, to be associated with longer sleep duration and improved sleep quality (Peach et al., 2018).

An increased level of perceived barriers toward going to bed on time was most strongly associated with decreased levels of general sleep quality, increased levels of daytime sleepiness, and increased SOLs. Especially the observed increases in SOLs (6.6 min on school days and 4.8 min on free days) that were associated with increases in perceived barriers are quite large when considering the mean SOL in the current sample (26 min on school days and 20 min on free days at T0, and 22 min on school days and 18 min on free days at T1). A larger variety of barriers was assessed in this study: time constraints (hobbies in the evening, no time for relaxation), perceived sleep difficulties (falling asleep, wake after sleep onset, waking too early), school stress (including homework in the evening), worries, fear of missing out (related to TV programs as well as social media conversations), and perceiving sleeping early as boring. Some of these perceived barriers have already been investigated. For example, the biopsychosocial contextual model of adolescent sleep suggests that mental health and academic factors belong to the main psychosocial factors influencing adolescent sleep (Becker et al., 2015). Moreover, previous longitudinal research has shown that emotional and behavioral difficulties predicted current and future sleep problems in adolescence (Kortesoja et al., 2020). Lastly, especially social-media related screen use has been shown to negatively affect adolescent sleep (Mireku et al., 2019). Future studies should further investigate which of these barriers are most strongly associated with sleep parameters. Interestingly, perceived barriers were found to be associated with all sleep quality parameters, but not with sleep duration. An explanation might be that some of the perceived barriers were about sleep difficulties, which are similar to some parameters of sleep quality. For example, the perceived barrier of not being able to directly fall asleep is reflected in SOL, and the perceived barrier of waking up during the night is reflected in general sleep quality. However, correlations between the psychosocial determinant perceived barriers and SOL on school days and free days (r_T0_ = 0.23, r_T1_ = 0.15, and r_T0_ = 0.16, r_T1_ = 0.12, respectively) and general sleep quality (r_T0_ = −0.51, r_T1_ = −0.34) were small to moderate (Akoglu, 2018). Altogether, the current findings illustrate that psychosocial factors impeding adolescent sleep quality might be related to psychosocial wellbeing and certain emotional states, and underscore the need to look at adolescent sleep in relation to mental health.

Previous research has found positive psychosocial factors like increased self-efficacy to be a buffer for negative psychosocial factors such as stress (Mikkelsen et al., 2020). This is reflected in the current findings, as an increased level of self-efficacy was found to be significantly associated with improved levels of sleep quality parameters, although the associations were rather weak. Considering that self-efficacy and perceived barriers were both associated with sleep quality parameters, we might speculate that increased levels of self-efficacy might be a buffer to overcome perceived barriers and aid healthy sleep in adolescence. As adolescents reported low levels of self-efficacy when it came to improving their sleep behavior in previous studies (Vandendriessche et al., 2022), there seems to be room for improvement, indicating that future interventions aiming to improve adolescent sleep could focus on increasing self-efficacy. Especially in the developmental context of adolescence, self-efficacy might be a valuable target, as the belief to be able to do things might relate to adolescents’ increased need for autonomy.

Altogether, the discussed findings suggest that psychosocial determinants identified by several leading behavior change theories can be applied to adolescent sleep. Specifically, perceived parental support (i.e., bedtime rules) and adolescents’ attitudes might be relevant to adolescent sleep duration, while perceived barriers and self-efficacy might be relevant for sleep quality. The current study does not allow any firm explanations on why these psychosocial determinants were differentially related to sleep duration and sleep quality parameters, which underlines the complexity of adolescent sleep. This complexity is also reflected in findings of other studies which indicated that some sleep parameters, such as sleep duration and efficiency, are explained by genetic factors, while others, such as sleep midpoint variability, are explained to a greater extent by environmental factors (Breitenstein et al., 2021). Sleep parameters which are more explained by environmental factors might be more appropriate targets for interventions. Especially for these sleep parameters, it might be interesting to further investigate the most strongly associated psychosocial determinants. However, it should also be noted that sleep duration and sleep quality are interrelated, which is reflected in them sharing genetic factors (Breitenstein et al., 2021). This, in turn, might make it difficult for sleep interventions to specifically target either sleep duration or quality.

While the observed increases in sleep duration are noteworthy, beta coefficients indicate that the observed associations were rather weak. A possible explanation might be that intention was not assessed in the current study. Behavior change theories postulate that intention to engage in a behavior is an important step between the psychosocial determinants of the behavior and actual performance of the behavior (Ajzen, 1991; Fishbein & Ajzen, 2011; de Vries et al. (1988)). Applied to sleep, adolescents might intend to go to bed on time but not do so, which could be reflected in shorter sleep durations; or they might intend to improve sleep hygiene behavior but not do so, which could be reflected in reduced sleep quality. This is also referred to as the intention-behavior gap (Sheeran & Webb, 2016). It remains the question whether stronger associations would be observed when including changes in adolescents’ intention to perform healthy sleep behavior in the analyses. Theories propose that perceived barriers can hinder the performance of actual behavior even if the intention to perform that behavior is present, while perceived support can facilitate the performance of actual behavior whilst the intention to perform that behavior is present. Therefore, perceived barriers and perceived support influence the pathway from intention to actual behavior, which might explain why these psychosocial determinants were most strongly related with sleep parameters in the current study.

This study had both strengths and limitations. Strengths are its longitudinal two-wave panel design and the large sample size. Moreover, CIBER (Crutzen et al., 2017) was used as an additional visual approach to establish determinant relevance of several psychosocial determinants simultaneously, and confirmed results from linear regression analyses. However, there are some limitations that should be addressed as well. First, even though validated measures were used, sleep was measured using self-report. Self-report might represent time spent in bed, instead of real time spent asleep. This might have biased results for sleep duration especially, and calls for objective measures of sleep. Nevertheless, recent findings indicate that objective and subjective measures might assess different sleep parameters (Breitenstein et al., 2021), suggesting that future studies could combine objective and subjective measures to assess sleep. Secondly, the measure of sleep duration was calculated using the midpoint method to recode the answer options, which is not an exact measure. Third, both psychosocial determinants and sleep parameters were assessed using a questionnaire, which might have led to common method variance problems. Another limitation relates to the assessment of psychosocial determinants. While the questionnaire that was co-created with a group of adolescents was tested for reliability and validity in a small sample, no psychometric study exists to confirm its robustness. As reliability estimates are quite low, future research should further investigate how to best assess psychosocial determinants of sleep in adolescents. Lastly, the items concerning perceived barriers were summed to have an overall score on the level of barriers adolescents perceive, however, future research could examine them separately. This might be especially important for choosing the most appropriate behavior change techniques when developing future healthy sleep interventions. For example, positive or negative arousal states might be addressed by emotion-regulation techniques, while fear of missing out related to social media use might be better addressed by specifying action plans to reduce screen use before bedtime (Gollwitzer & Sheeran, 2006). Finally, it should be noted that the current results are limited to the context of Flemish Belgium and might not be generalizable to other age- or sociocultural groups.

## Conclusion

Psychosocial determinants identified by several behavior change theories have been proposed to explain a variety of health behaviors in adolescence. However, little research has investigated these psychosocial determinants in relation to adolescent sleep behavior. To bridge this research gap, the current study explored whether changes in psychosocial determinants of sleep were associated with changes in sleep duration and sleep quality parameters in adolescents. Increased levels of bedtime rules (i.e., perceived parental support) were found to be most strongly associated with improved levels of sleep duration, while an increased level of perceived barriers toward healthy sleep and going to bed on time was found to be most strongly associated with improved sleep quality parameters. The current findings indicate that parental support might positively influence adolescent sleep health, even though adolescence is a phase in which reliance on parents might decrease and the need for autonomy and independence might increase. Moreover, an increased perception of barriers of healthy sleep is related to less favorable changes in sleep. Finally, the current results indicate that a positive attitude toward healthy sleep might improve adolescent sleep health. As the current study cannot infer causality, future research is needed to confirm these results. The identification of the most important psychosocial determinants of sleep is a first step and can contribute to the development of healthy sleep interventions.

### Supplementary Information

Appendix 1

Appendix 2

Appendix 3

Appendix 4
