# Supplementary material for: Psychosocial Determinants of Sleep Behavior and Healthy Sleep Among Adolescents: A Two-Wave Panel Study
Source: J Youth Adolesc. 2023 Sep 25;53(2):360–73. doi: 10.1007/s10964-023-01866-8 (PMC10764366; doi:10.1007/s10964-023-01866-8)
Supplement: Supplementary file 1 — Appendix 1 [file 10964_2023_1866_MOESM1_ESM.docx]

**Appendix 1**

*Determinant items and Cronbach’s alphas (T0 and T1) per subscale*

| **Knowledge** *(Cronbach’s alpha T0 = 0.67, Cronbach’s alpha T1 = 0.67)* |
| --- |
| 1.You can make up for little sleep by drinking caffeinated soft drinks (e.g., Coca Cola, Red Bull, Nalu, Monster…) or coffee. |
| 2.Drinking alcohol (even in small amounts) helps you to sleep well. |
| 3.Sitting a lot during the day makes you sleep better at night. |
| 4.Sporting before bedtime (± 1 hour) makes falling asleep harder. |
| 5.Using your smartphone/tablet/laptop just before bedtime makes falling asleep more difficult. |
| 6.If you don’t sleep enough now, you can experience health problems as an adult. |
| 7.Sleep deprivation can make you feel less good. |
| 8.Getting enough sleep can make you tan faster. |
| 9.Sleep deprivation negatively influences reaction times. |
| 10.Sleep deprivation negatively influences your concentration. |
| 11.Sleep deprivation can cause you to have more frequent nosebleeds. |
| 12.Sleep deprivation can cause you to gain weight. |
| 13.Sufficient sleep ensures that your body and brain can recover from the past day. |
| 14.How many hours of sleep does someone of your age group need, according to you? |

| **Attitude** *(Cronbach’s alpha T0 = 0.71, Cronbach’s alpha T1 = 0.78)* |
| --- |
| 1.I find it important to sleep sufficient and sufficiently well. |
| 2.I find it important to go to bed on time. |

| **Perceived advantages** *(Cronbach’s alpha T0= 0.81, Cronbach’s alpha T1= 0.82)* |
| --- |
| An advantage of going to bed early is… |
| 1.…that I sleep sufficiently. |
| 2.…that I am in a good mood the next day. |
| 3.…that I am more concentrated at school the next day. |
| 4.…that I get out of bed easier in the morning. |
| 5.…that I don’t feel tired during the day. |
| 6.…that I get sick less easily. |
| 7.…that I have more energy during the day. |
| 8.…that I do better at school. |
| 9.…that I feel more energized and better. |

| **Self-efficacy** *(Cronbach’s alpha T0 = 0.80, Cronbach’s alpha T1 = 0.74)* |
| --- |
| I think that I am able to go to bed on time… |
| 1.…even if I miss a specific TV program. |
| 2.…even if I have to do a lot of school work. |
| 3.…even if I miss specific conversations on social media. |
| 4.…even if my parents go to bed later. |
| 5.…even if my brothers and sisters go to bed later. |
| 6.…even if I miss meetings with friends (for example game nights, activities in a youth center) |
| 7.…even if I am used to go to bed later. |
| 8.…even if I don’t feel like it. |

| **Perceived norms peers** *(Cronbach’s alpha T0 = 0.71, Cronbach’s alpha T1 = 0.66)* |
| --- |
| My best friends… |
| 1.…go to bed on time. (modeling) |
| 2.…sleep sufficiently. (modeling) |
| 3.…sleep well. (modeling) |
| 4.…use their smartphone/tablet/laptop when they lie in bed. (modeling) |
| 5.…think that social media is more important than getting enough sleep. (perceived peer norm) |
| 6.…think that finishing their school work is more important than getting enough sleep. (perceived peer norm) |
| 7.…think that watching specific TV programs is more important than getting enough sleep. (perceived peer norm) |

| **Perceived norms parents** *(Cronbach’s alpha T0 = 0.65, Cronbach’s alpha T1 = 0.65)* |
| --- |
| My parents… |
| 1.…go to bed on time. (modeling) |
| 2.…sleep sufficiently. (modeling) |
| 3.…sleep well. (modeling) |
| 4.…use their smartphone/tablet/laptop when they lie in bed. (modeling) |
| 5.…think that social media is more important than getting enough sleep. (perceived parental norm) |
| 6.…think that finishing their school work is more important than getting enough sleep. (perceived parental norm) |
| 7.…think that watching specific TV programs is more important than getting enough sleep. (perceived parental norm) |
| …find it important that I get enough sleep. (perceived parental norm related to the adolescent) |

| **Perceived barriers** *(Cronbach’s alpha T0 = 0.67, Cronbach’s alpha T1= 0.69)* |
| --- |
| What prevents me from going to bed on time is… |
| 1.…that going to bed on time is boring. |
| 2.…that I still lie awake. |
| 3.…that I want to see specific TV programmes. |
| 4.…that I want to follow specific conversations on social media (for example on Facebook, Snapchat, Instagram, …) |
| 5.…that I have to/want to finish my homework. |
| 6.…that I wake up too early in the morning. |
| 7.…that I wake up during the night. |
| 8.…that I don’t have sufficient time to relax before bedtime. |
| 9.…that I have hobby’s in the evening. |
| 10.…that I lie awake worrying. |
| 11.…that I have too much stress caused by my school work. |

| **Perceived parental support** *(Cronbach’s alpha T0 = 0.62 , Cronbach’s alpha T1 = 0.69)* |
| --- |
| 1.My parents encourage me to go to bed on time. (encouragement) |
| 2.On school days, I have a set bedtime, imposed by my parents. (bedtime rules) |
| 3.On free days, I have a set bedtime, imposed by my parents. (bedtime rules) |
