# Supplementary material for: Psychosocial Determinants of Sleep Behavior and Healthy Sleep Among Adolescents: A Two-Wave Panel Study
Source: J Youth Adolesc. 2023 Sep 25;53(2):360–73. doi: 10.1007/s10964-023-01866-8 (PMC10764366; doi:10.1007/s10964-023-01866-8)
Supplement: Supplementary file 2 — Appendix 2 [file 10964_2023_1866_MOESM2_ESM.docx]

**Appendix 2**

*CIBER results*

The following plots show determinants of sleep behavior, their distributions and means in the left panel, and their association to sleep behaviors in the right panel. The fill color of the diamonds in the left panel indicates the determinant means, with blue denoting determinants in the middle of the scale. The fill color of the diamonds on the right indicates association strength and direction. The redder the fill color, the stronger and more negative the associations are. The greener the fill color, the stronger and more positive the associations are. The greyer the fill color, the weaker the associations. Confidence intervals are depicted at the top of the figures. Overall results show that determinant means are distributed around the middle of the scale, indicating room for improvement for all determinants, as participants do not have the desired values, yet (as for example a very positive attitude towards healthy sleep, or a very low number of perceived barriers towards going to bed on time). Moreover, overall associations are quite weak, indicated by the mostly greyish diamond fill colors. In the tables, CIBER results are compared to the results of the linear models.

**Figure 2.1**

*CIBER results. Means and associations of sleep quantity on school days and free days with determinants*


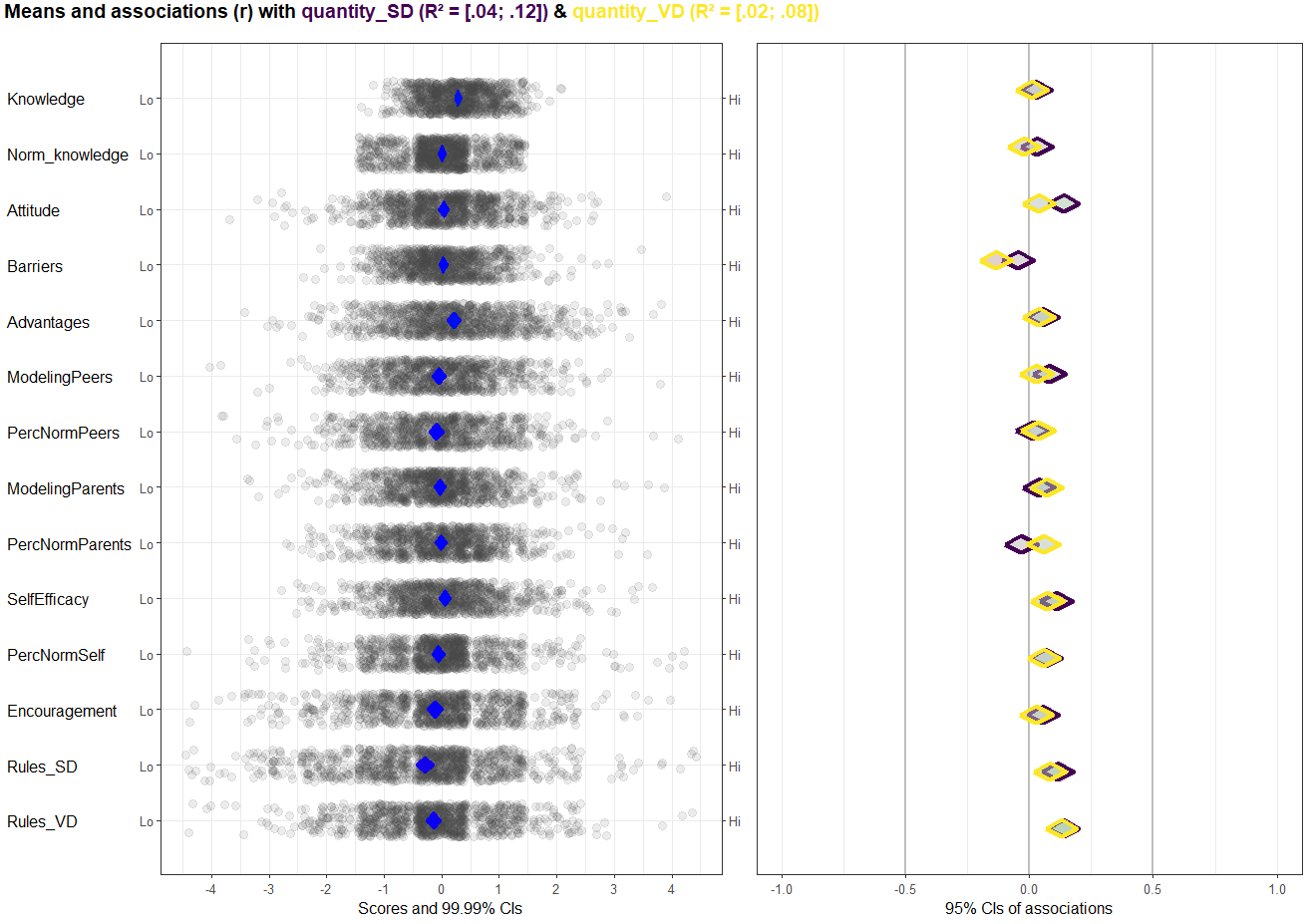


**Table 2.1**

*Comparing results sleep quantity school days*

| CIBER results school days | Linear model results school days |
| --- | --- |
| Attitude (+)^1^ | Attitude (+)^1^ |
| Bedtime rules as imposed by parents (+)^1^ | Bedtime rules as imposed by parents (+)^1^ |
| Barriers (-)^2^ | - ^3^ |
| Perceived parental norm (-)^2^ | - ^3^ |
| - ^3^ | Norm-knowledge (+)^1^ |
| - ^3^ | Peer behavior (+)^1^ |

^1^A + indicates a positive association or a positive effect. ^2^A – indicates a negative association or negative effect. ^3^If the determinant is not mentioned (-), this indicates that there was no significant effect or notable association.

**Table 2.2**

*Comparing results sleep quantity free days*

| CIBER results free days | Linear model results free days |
| --- | --- |
| Bedtime rules as imposed by parents (+)^1^ | Bedtime rules as imposed by parents (+)^1^ |
| Barriers (-)^2^ | - ^3^ |
| - ^3^ | Perceived advantages (+)^1^ |

^1^A + indicates a positive association or a positive effect. ^2^A – indicates a negative association or negative effect. ^3^If the determinant is not mentioned (-), this indicates that there was no significant effect or notable association.

**Figure 2.2**

*CIBER results. Means and associations of general sleep quality as assessed with the s-ASWS with determinants*


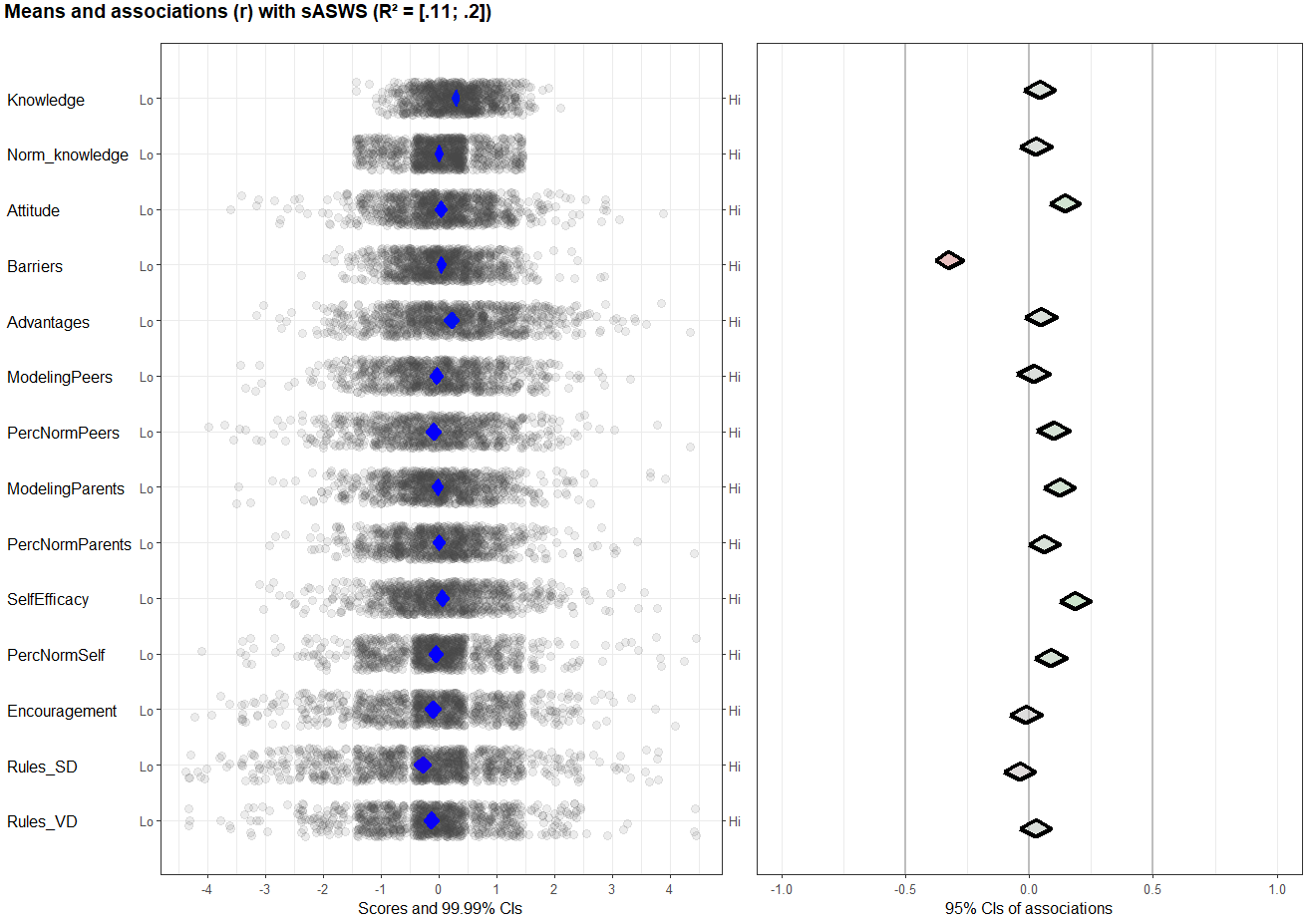


**Table 2.3**

*Comparing results general sleep quality*

| CIBER results general sleep quality | Linear model results general sleep quality |
| --- | --- |
| Barriers (-)^1^ | Barriers(-)^1^ |
| Attitude (+)^2^ | Attitude (+)^2^ |
| Self-efficacy (+)^2^ | Self-efficacy (+)^2^ |

^1^A - indicates a negative association or a negative effect. ^2^A + indicates a positive association or positive effect.

**Figure 2.3**

*CIBER results. Means and associations of sleep quality as assessed with the PDSS with determinants*


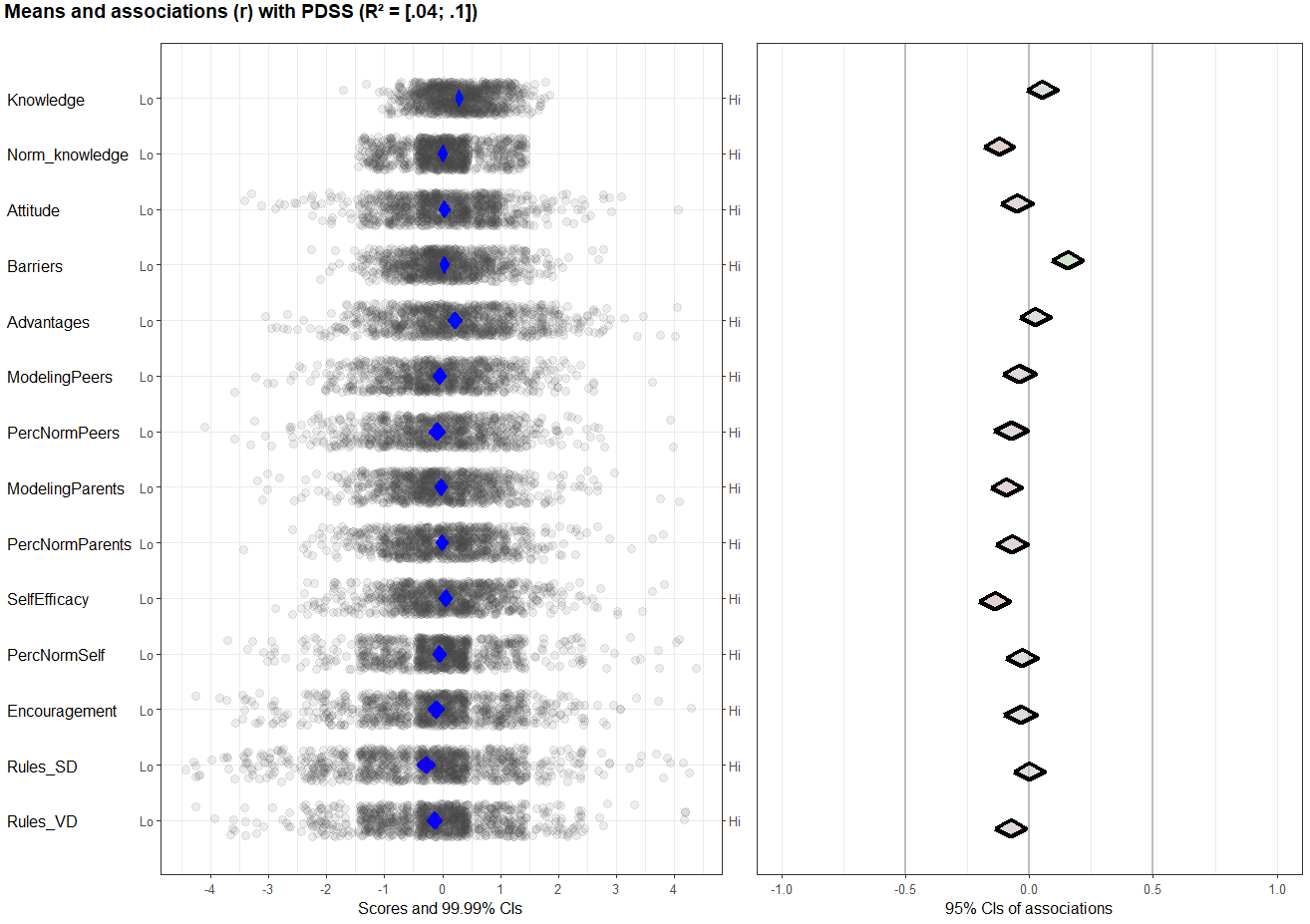


**Table 2.4**

*Comparing results for daytime sleepiness*

| CIBER results daytime sleepiness | Linear model results daytime sleepiness |
| --- | --- |
| Barriers (+)^1^ | Barriers (+)^1^ |
| Norm knowledge (-)^2^ | - ^3^ |
| Self-efficacy (-)^2^ | Self-efficacy (-)^2^ |
| - ^3^ | Parental behavior (-)^2^ |

^1^A + indicates a positive association or a positive effect. ^2^A – indicates a negative association or negative effect. ^3^If the determinant is not mentioned (-), this indicates that there was no significant effect or notable association.

**Figure 2.4**

*CIBER results. Means and associations of sleep quality as indicated by sleep onset latency on school days and free days with determinants*


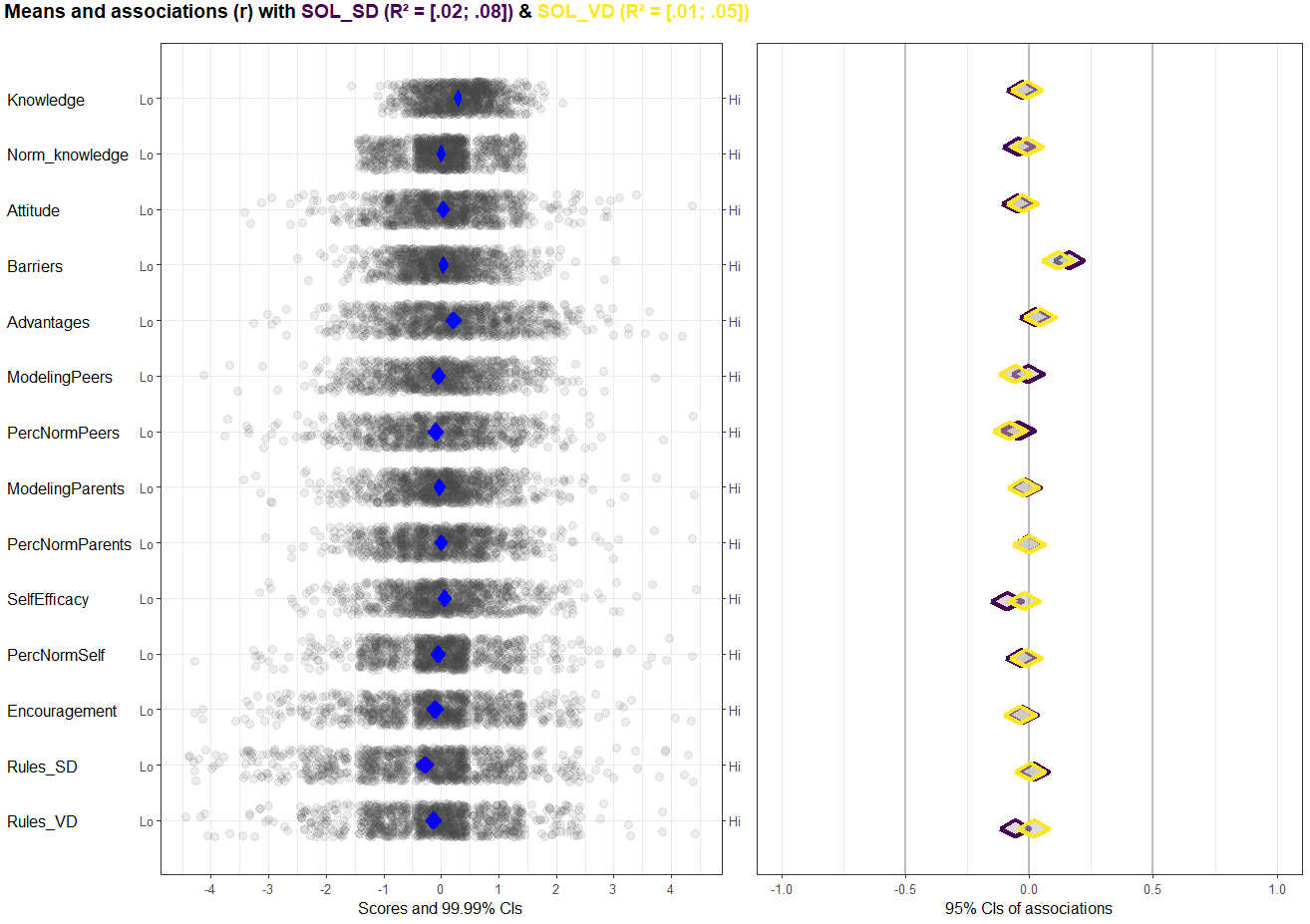


**Table 2.5**

*Comparing results SOL school days*

| CIBER results SOL school days | Linear model results SOL school days |
| --- | --- |
| Barriers (+)^1^ | Barriers (+)^1^ |
| Self-efficacy (-)^2^ | - ^3^ |

^1^A + indicates a positive association or a positive effect. ^2^A – indicates a negative association or negative effect. ^3^If the determinant is not mentioned (-), this indicates that there was no significant effect or notable association.

**Table 2.6**

*Comparing results SOL free days*

| CIBER results SOL free days | Linear model results SOL free days |
| --- | --- |
| Barriers (+)^1^ | Barriers (+)^1^ |
| - ^3^ | Perceived norm parents (+)^1^ |

^1^A + indicates a positive association or a positive effect. ^3^If the determinant is not mentioned (-), this indicates that there was no significant effect or notable association.
