# Supplementary material for: Psychosocial Determinants of Sleep Behavior and Healthy Sleep Among Adolescents: A Two-Wave Panel Study
Source: J Youth Adolesc. 2023 Sep 25;53(2):360–73. doi: 10.1007/s10964-023-01866-8 (PMC10764366; doi:10.1007/s10964-023-01866-8)
Supplement: Supplementary file 3 — Appendix 3 [file 10964_2023_1866_MOESM3_ESM.docx]

**Table 3.1**

*Correlations for sleep parameters*

| Variable | 1 | 2 | 3 | 4 | 5 | 6 | 7 | 8 | 9 | 10 | 11 | 12 |
| --- | --- | --- | --- | --- | --- | --- | --- | --- | --- | --- | --- | --- |
| 1. Sleep duration school days T0 | — |  |  |  |  |  |  |  |  |  |  |  |
| 2. Sleep duration free days T0 | .31 | — |  |  |  |  |  |  |  |  |  |  |
| 3. General sleep quality T0 | .36 | .20 | — |  |  |  |  |  |  |  |  |  |
| 4. Daytime sleepiness T0 | -.24 | .08 | -.39 | — |  |  |  |  |  |  |  |  |
| 5. SOL school days T0 | -.41 | -.14 | -.35 | .15 | — |  |  |  |  |  |  |  |
| 6. SOL free days T0 | -.29 | .20 | -.26 | .09 | .66 | — |  |  |  |  |  |  |
| 7. Sleep duration school days T1 | .56 | .18 | .26 | -.20 | -.23 | .22 | — |  |  |  |  |  |
| 8. Sleep duration free days T1 | .21 | .51 | .15 | .05 | -.05 | -.05 | .30 | — |  |  |  |  |
| 9. General sleep quality T1 | .24 | .14 | .53 | -.28 | -.27 | -.24 | .33 | .18 | — |  |  |  |
| 10. Daytime sleepiness T1 | -.23 | .10 | -.25 | .62 | .10 | .07 | -.24 | .09 | .39 | — |  |  |
| 11. SOL school days T1 | -.18 | -.10 | -.21 | .08 | .56 | .50 | -.31 | -.12 | .30 | .12 | — |  |
| 12. SOL free days T1 | -.14 | -.10 | -.15 | .01 | .41 | .45 | -.20 | -.19 | .27 | .08 | .67 | — |

**Table 3.2**

*Correlations for psychosocial determinants*

| Variable | 1 | 2 | 3 | 4 | 5 | 6 | 7 | 8 | 9 | 10 | 11 | 12 | 13 | 14 | 15 | 16 | 17 | 18 | 19 | 20 | 21 | 22 | 23 | 24 | 25 | 26 | 27 | 28 |
| --- | --- | --- | --- | --- | --- | --- | --- | --- | --- | --- | --- | --- | --- | --- | --- | --- | --- | --- | --- | --- | --- | --- | --- | --- | --- | --- | --- | --- |
| 1. Knowledge T0 | — |  |  |  |  |  |  |  |  |  |  |  |  |  |  |  |  |  |  |  |  |  |  |  |  |  |  |  |
| 2. Norm knowledge T0 | .02 | — |  |  |  |  |  |  |  |  |  |  |  |  |  |  |  |  |  |  |  |  |  |  |  |  |  |  |
| 3. Attitude T0 | .16 | .12 | — |  |  |  |  |  |  |  |  |  |  |  |  |  |  |  |  |  |  |  |  |  |  |  |  |  |
| 4. Barriers T0 | -.07 | -.03 | -.23 | — |  |  |  |  |  |  |  |  |  |  |  |  |  |  |  |  |  |  |  |  |  |  |  |  |
| 5. Perceived  advantages T0 | .25 | .04 | .29 | -.03 | — |  |  |  |  |  |  |  |  |  |  |  |  |  |  |  |  |  |  |  |  |  |  |  |
| 6. Modeling peers T0 | .03 | .08 | .23 | -.19 | .01 | — |  |  |  |  |  |  |  |  |  |  |  |  |  |  |  |  |  |  |  |  |  |  |
| 7. Perceived norm  peers T0 | .03 | .00 | .12 | -.26 | -.01 | .30 | — |  |  |  |  |  |  |  |  |  |  |  |  |  |  |  |  |  |  |  |  |  |
| 8. Modeling parents T0 | .02 | .05 | .20 | -.16 | .08 | .17 | .10 | — |  |  |  |  |  |  |  |  |  |  |  |  |  |  |  |  |  |  |  |  |
| 9. Perceived norm parents T0 | -.01 | .06 | .16 | -.24 | .01 | .11 | .22 | .38 | — |  |  |  |  |  |  |  |  |  |  |  |  |  |  |  |  |  |  |  |
| 10. Self-efficacy T0 | .13 | .13 | .39 | -.31 | .14 | .24 | .15 | .20 | .15 | — |  |  |  |  |  |  |  |  |  |  |  |  |  |  |  |  |  |  |
| 11. Perceived norm parents related to adolescent behavior T0 | .03 | .07 | .30 | -.10 | .14 | .14 | .02 | .18 | .12 | .22 | — |  |  |  |  |  |  |  |  |  |  |  |  |  |  |  |  |  |
| 12. Parental encouragement T0 | .01 | -.01 | .20 | -.05 | .13 | .08 | -.01 | .14 | .11 | .15 | .49 | — |  |  |  |  |  |  |  |  |  |  |  |  |  |  |  |  |
| 13. Bedtime rules school days T0 | .08 | .08 | .15 | -.03 | .05 | .16 | .03 | .13 | .09 | .14 | .24 | .29 | — |  |  |  |  |  |  |  |  |  |  |  |  |  |  |  |
| 14. Bedtime rules free days T0 | .03 | .09 | .23 | -.06 | .04 | .22 | .06 | .13 | .08 | .16 | .20 | .22 | .53 | — |  |  |  |  |  |  |  |  |  |  |  |  |  |  |
| 15. Knowledge T1 | .05 | .08 | .19 | -.08 | .04 | .13 | .09 | .04 | .09 | .13 | .12 | .11 | .03 | .08 | — |  |  |  |  |  |  |  |  |  |  |  |  |  |
| 16. Norm knowledge T1 | .03 | .18 | .09 | -.00 | .05 | .03 | .08 | .02 | .00 | .04 | .05 | .03 | .07 | .07 | .06 | — |  |  |  |  |  |  |  |  |  |  |  |  |
| 17. Attitude T1 | .06 | .07 | .48 | -.20 | .21 | .16 | .14 | .12 | .15 | .27 | .23 | .16 | .09 | .17 | .22 | .18 | — |  |  |  |  |  |  |  |  |  |  |  |
| 18. Barriers T1 | -.03 | -.02 | -.14 | .46 | -.01 | -.10 | -.15 | -.09 | -.14 | -.21 | -.09 | -.07 | -.06 | -.05 | -.07 | -.10 | -.20 | — |  |  |  |  |  |  |  |  |  |  |
| 19. Perceived advantages T1 | .07 | .08 | .25 | -.06 | .29 | .05 | -.01 | .05 | .05 | .18 | .16 | .17 | .06 | .12 | .26 | .13 | .40 | -.03 | — |  |  |  |  |  |  |  |  |  |
| 20. Modeling peers T1 | .12 | .07 | .19 | -.09 | .01 | .37 | .15 | .13 | .12 | .17 | .10 | -.00 | .10 | .14 | .07 | .06 | .14 | -.09 | .04 | — |  |  |  |  |  |  |  |  |
| 21. Perceived norm peers T1 | .08 | .01 | .05 | -.15 | .01 | .09 | .23 | .11 | .15 | .12 | .06 | .03 | .05 | .04 | .02 | .03 | .02 | -.28 | -.10 | .27 | — |  |  |  |  |  |  |  |
| 22. Modeling parents T1 | .05 | .06 | .07 | -.08 | .00 | .08 | .05 | .47 | .22 | .10 | .09 | .10 | .12 | .10 | .08 | .06 | .15 | -.16 | .14 | .10 | .05 | — |  |  |  |  |  |  |
| 23. Perceived norm parents T1 | -.02 | .06 | .15 | -.13 | .07 | .05 | .11 | .28 | .41 | .14 | .16 | .12 | .07 | .04 | .14 | .03 | .16 | -.24 | .10 | .05 | .19 | .36 | — |  |  |  |  |  |
| 24. Self-efficacy T1 | .14 | .06 | .26 | -.27 | .14 | .12 | .15 | .08 | .12 | .37 | .16 | .08 | .04 | .09 | .15 | .07 | .32 | -.31 | .26 | .20 | .18 | .16 | .20 | — |  |  |  |  |
| 25. Perceived norm parents related to adolescent behavior T1 | .06 | .07 | .15 | -.04 | .10 | .12 | .09 | .09 | .08 | .07 | .32 | .27 | .22 | .18 | .18 | .08 | .27 | -.11 | .26 | .07 | .05 | .20 | .20 | .19 | — |  |  |  |
| 26. Parental encouragement T1 | -.00 | .06 | .12 | -.03 | .07 | .12 | -.01 | .09 | .09 | .07 | .27 | .36 | .28 | .24 | .18 | .05 | .21 | -.06 | .22 | .10 | .06 | .16 | .13 | .18 | .49 | — |  |  |
| 27. Bedtime rules school days T1 | .07 | .07 | .11 | -.05 | -.01 | .14 | .10 | .06 | .07 | .07 | .13 | .20 | .55 | .43 | .06 | .06 | .04 | -.04 | .04 | .19 | .11 | .11 | .04 | .07 | .28 | .37 | — |  |
| 28. Bedtime rules free days T1 | .04 | .04 | .14 | -.05 | .02 | .15 | .05 | .07 | .07 | .09 | .17 | .21 | .39 | .55 | .09 | .08 | .13 | -.08 | .09 | .17 | .09 | .11 | .05 | .12 | .22 | .30 | .58 | — |

**Table 3.3**

*Correlations for psychosocial determinants and sleep parameters*

| Variable | 1 | 2 | 3 | 4 | 5 | 6 | 7 | 8 | 9 | 10 | 11 | 12 | 13 | 14 | 15 | 16 | 17 | 18 | 19 | 20 | 21 | 22 | 23 | 24 | 25 | 26 | 27 | 28 | 29 | 30 | 31 | 32 | 33 | 34 | 35 | 36 | 37 | 38 | 39 | 40 |
| --- | --- | --- | --- | --- | --- | --- | --- | --- | --- | --- | --- | --- | --- | --- | --- | --- | --- | --- | --- | --- | --- | --- | --- | --- | --- | --- | --- | --- | --- | --- | --- | --- | --- | --- | --- | --- | --- | --- | --- | --- |
| 1. Sleep duration school days T0 | — |  |  |  |  |  |  |  |  |  |  |  |  |  |  |  |  |  |  |  |  |  |  |  |  |  |  |  |  |  |  |  |  |  |  |  |  |  |  |  |
| 2. Sleep duration free days T0 | .31 | — |  |  |  |  |  |  |  |  |  |  |  |  |  |  |  |  |  |  |  |  |  |  |  |  |  |  |  |  |  |  |  |  |  |  |  |  |  |  |
| 3. general sleep quality T0 | .36 | .20 | — |  |  |  |  |  |  |  |  |  |  |  |  |  |  |  |  |  |  |  |  |  |  |  |  |  |  |  |  |  |  |  |  |  |  |  |  |  |
| 4. daytime sleepiness T0 | -.24 | .08 | -.39 | — |  |  |  |  |  |  |  |  |  |  |  |  |  |  |  |  |  |  |  |  |  |  |  |  |  |  |  |  |  |  |  |  |  |  |  |  |
| 5. SOL school days T0 | -.41 | -.14 | -.35 | .15 | — |  |  |  |  |  |  |  |  |  |  |  |  |  |  |  |  |  |  |  |  |  |  |  |  |  |  |  |  |  |  |  |  |  |  |  |
| 6. SOL free days T0 | -.29 | -.20 | -.26 | .09 | .66 | — |  |  |  |  |  |  |  |  |  |  |  |  |  |  |  |  |  |  |  |  |  |  |  |  |  |  |  |  |  |  |  |  |  |  |
| 7. sleep duration school days T1 | .56 | .18 | .26 | -.20 | -.23 | -.22 | — |  |  |  |  |  |  |  |  |  |  |  |  |  |  |  |  |  |  |  |  |  |  |  |  |  |  |  |  |  |  |  |  |  |
| 8. sleep duration free days T1 | .21 | .51 | .15 | .05 | -.05 | -.05 | .30 | — |  |  |  |  |  |  |  |  |  |  |  |  |  |  |  |  |  |  |  |  |  |  |  |  |  |  |  |  |  |  |  |  |
| 9. general sleep quality T1 | .24 | .14 | .53 | -.28 | -.27 | -.24 | .33 | .18 | — |  |  |  |  |  |  |  |  |  |  |  |  |  |  |  |  |  |  |  |  |  |  |  |  |  |  |  |  |  |  |  |
| 10. daytime sleepiness T1 | -.23 | .10 | -.25 | .62 | .10 | .07 | -.24 | .09 | -.39 | — |  |  |  |  |  |  |  |  |  |  |  |  |  |  |  |  |  |  |  |  |  |  |  |  |  |  |  |  |  |  |
| 11. SOL school days T1 | -.18 | -.10 | -.21 | .08 | .56 | .50 | -.31 | -.12 | -.30 | .01 | — |  |  |  |  |  |  |  |  |  |  |  |  |  |  |  |  |  |  |  |  |  |  |  |  |  |  |  |  |  |
| 12. SOL free days T1 | -.14 | -.10 | -.15 | .01 | .41 | .45 | -.20 | -.19 | -.27 | .08 | .67 | — |  |  |  |  |  |  |  |  |  |  |  |  |  |  |  |  |  |  |  |  |  |  |  |  |  |  |  |  |
| 13. Knowledge T0 | .08 | -.03 | .11 | -.04 | .00 | -.00 | .05 | -.01 | .10 | -.09 | .03 | .01 | — |  |  |  |  |  |  |  |  |  |  |  |  |  |  |  |  |  |  |  |  |  |  |  |  |  |  |  |
| 14. Norm knowledge T0 | .12 | -.02 | .07 | -.07 | -.02 | .00 | .11 | .02 | .07 | .00 | -.01 | -.01 | .02 | — |  |  |  |  |  |  |  |  |  |  |  |  |  |  |  |  |  |  |  |  |  |  |  |  |  |  |
| 15. Attitude T0 | .31 | .17 | .32 | -.25 | -.02 | -.01 | .23 | .16 | .17 | -.17 | .02 | .02 | .16 | .13 | — |  |  |  |  |  |  |  |  |  |  |  |  |  |  |  |  |  |  |  |  |  |  |  |  |  |
| 16. Perceived barriers T0 | -.26 | -.08 | -.51 | .32 | .23 | .15 | -.25 | -.08 | -.34 | .28 | .13 | .06 | -.07 | -.03 | -.23 | — |  |  |  |  |  |  |  |  |  |  |  |  |  |  |  |  |  |  |  |  |  |  |  |  |
| 17. Perceived advantaged T0 | .11 | .05 | .12 | -.08 | -.07 | -.05 | .07 | .07 | .09 | -.08 | -.04 | -.04 | .25 | .04 | .29 | -.03 | — |  |  |  |  |  |  |  |  |  |  |  |  |  |  |  |  |  |  |  |  |  |  |  |
| 18. Modeling peers T0 | .21 | .10 | .19 | -.19 | -.01 | -.06 | .15 | .08 | .12 | -.18 | -.01 | .01 | .03 | .08 | .23 | -.19 | .01 | — |  |  |  |  |  |  |  |  |  |  |  |  |  |  |  |  |  |  |  |  |  |  |
| 19. Perceived norms peers T0 | .17 | .06 | .15 | -.17 | -.05 | -.04 | .09 | .04 | .05 | -.12 | .00 | .03 | .04 | .00 | .12 | -.26 | -.01 | .30 | — |  |  |  |  |  |  |  |  |  |  |  |  |  |  |  |  |  |  |  |  |  |
| 20. Modeling parents T0 | .12 | .09 | .22 | -.22 | -.07 | -.04 | .11 | .07 | .15 | -.16 | -.05 | -.02 | .02 | .05 | .20 | -.16 | .08 | .17 | .10 | — |  |  |  |  |  |  |  |  |  |  |  |  |  |  |  |  |  |  |  |  |
| 21. Perceived norm parents T0 | .13 | .04 | .16 | -.18 | -.03 | -.01 | .12 | .01 | .11 | -.11 | -.01 | .01 | -.01 | .06 | .16 | -.24 | .01 | .11 | .22 | .40 | — |  |  |  |  |  |  |  |  |  |  |  |  |  |  |  |  |  |  |  |
| 22 Self-efficacy T0 | .25 | .10 | .32 | -.30 | -.04 | -.07 | .20 | .10 | .22 | -.24 | .03 | -.02 | .13 | .13 | .39 | -.31 | .14 | .24 | .15 | .20 | .15 | — |  |  |  |  |  |  |  |  |  |  |  |  |  |  |  |  |  |  |
| 23. Perceived norm parents related to adolescent behavior T0 | .20 | .09 | .10 | -.08 | -.02 | -.01 | .14 | .11 | .08 | -.14 | .04 | .02 | .03 | .07 | .30 | -.10 | .13 | .14 | .02 | .18 | .12 | .22 | — |  |  |  |  |  |  |  |  |  |  |  |  |  |  |  |  |  |
| 24. Parental encouragement T0 | .15 | .07 | .01 | -.03 | -.02 | -.03 | .10 | .08 | .03 | -.04 | .00 | -.03 | .01 | -.00 | .20 | -.05 | .13 | .08 | -.01 | .14 | .11 | .15 | .49 | — |  |  |  |  |  |  |  |  |  |  |  |  |  |  |  |  |
| 25. Bedtime rules school days T0 | .21 | .06 | -.00 | -.13 | .10 | .04 | .12 | .06 | .04 | -.14 | .09 | .02 | .08 | .08 | .15 | -.03 | .05 | .16 | .03 | .13 | .09 | .14 | .24 | .30 | — |  |  |  |  |  |  |  |  |  |  |  |  |  |  |  |
| 26. Bedtime rules free days T0 | .20 | .14 | .05 | -.15 | .05 | .06 | .10 | .09 | -.00 | -.12 | .07 | .02 | .03 | .09 | .22 | -.06 | .04 | .22 | .06 | .12 | .08 | .16 | .20 | .22 | .53 | — |  |  |  |  |  |  |  |  |  |  |  |  |  |  |
| 27. Knowledge T1 | .06 | -.00 | .06 | -.07 | .04 | .06 | .07 | .08 | .04 | .01 | .08 | .06 | .06 | .08 | .19 | -.08 | .04 | .13 | .09 | .04 | .09 | .13 | .12 | .11 | .03 | .08 | — |  |  |  |  |  |  |  |  |  |  |  |  |  |
| 28. Norm knowledge T1 | .10 | -.00 | -.02 | -.02 | .01 | -.01 | .14 | .08 | .03 | -.04 | -.04 | -.04 | .03 | .18 | .09 | -.00 | .05 | .03 | .08 | .02 | .00 | .04 | .05 | .03 | .07 | .07 | .06 | — |  |  |  |  |  |  |  |  |  |  |  |  |
| 29. Attitude T1 | .25 | .11 | .22 | -.16 | -.01 | -.03 | .30 | .16 | .24 | -.12 | -.01 | -.02 | .06 | .07 | .48 | -.20 | .21 | .16 | .14 | .12 | .15 | .27 | .23 | .16 | .10 | .17 | .22 | .18 | — |  |  |  |  |  |  |  |  |  |  |  |
| 30. Perceived barriers T1 | -.18 | .00 | -.36 | .25 | .16 | .12 | -.21 | -.10 | -.50 | .35 | .21 | .17 | -.03 | -.02 | -.15 | .46 | -.01 | -.10 | -.15 | -.09 | -.14 | -.21 | -.09 | -.07 | -.06 | -.05 | -.07 | -.10 | -.20 | — |  |  |  |  |  |  |  |  |  |  |
| 31. Perceived advantaged T1 | .21 | .09 | .17 | -.10 | -.15 | -.11 | .20 | .16 | .19 | -.08 | -.06 | -.02 | .07 | .08 | .25 | -.06 | .29 | .05 | -.01 | .05 | .05 | .18 | .16 | .17 | .06 | .12 | .26 | .13 | .40 | -.03 | — |  |  |  |  |  |  |  |  |  |
| 32. Modeling peers T1 | .13 | .01 | .12 | -.16 | .03 | .00 | .18 | .07 | .09 | -.16 | .02 | .01 | .12 | .07 | .19 | -.09 | .01 | .37 | .15 | .13 | .12 | .17 | .10 | -.00 | .10 | .14 | .08 | .06 | .14 | -.09 | .04 | — |  |  |  |  |  |  |  |  |
| 33. Perceived norms peers T1 | .04 | -.05 | .06 | -.13 | .03 | .01 | -.02 | .01 | .11 | -.14 | .03 | -.00 | .09 | .01 | .05 | -.15 | .01 | .09 | .23 | .11 | .15 | .12 | .06 | .03 | .05 | .04 | .02 | .03 | .02 | -.28 | -.09 | .27 | — |  |  |  |  |  |  |  |
| 34. Modeling parents T1 | .05 | .01 | .11 | -.16 | -.05 | -.05 | .10 | .08 | .19 | -.16 | -.03 | -.03 | .05 | .06 | .07 | -.08 | .00 | .08 | .05 | .47 | .22 | .10 | .09 | .10 | .12 | .10 | .08 | .06 | .15 | -.16 | .14 | .10 | .05 | — |  |  |  |  |  |  |
| 35. Perceived norm parents T1 | .08 | .04 | .10 | -.10 | .03 | .00 | .07 | .09 | .13 | -.10 | .03 | .03 | -.03 | .06 | .15 | -.13 | .07 | .05 | .11 | .28 | .41 | .14 | .16 | .12 | .07 | .04 | .14 | .03 | .16 | -.24 | .10 | .05 | .19 | .36 | — |  |  |  |  |  |
| 36. Self-efficacy T1 | .15 | .04 | .26 | -.24 | -.04 | -.05 | .19 | .09 | .31 | -.27 | -.03 | -.00 | .14 | .06 | .26 | -.27 | .14 | .12 | .15 | .08 | .12 | .37 | .16 | .08 | .04 | .09 | .15 | .07 | .32 | -.31 | .26 | .20 | .18 | .16 | .20 | — |  |  |  |  |
| 37. Perceived norm parents related to adolescent behavior T1 | .16 | .02 | .02 | -.08 | .03 | -.00 | .14 | .10 | .08 | -.15 | .02 | .00 | .06 | .07 | .15 | -.04 | .10 | .12 | .09 | .09 | .08 | .07 | .32 | .27 | .22 | .18 | .18 | .08 | .27 | -.11 | .26 | .07 | .05 | .20 | .20 | .19 | — |  |  |  |
| 38. Parental encouragement T1 | .16 | .04 | .04 | -.05 | .02 | .04 | .13 | .11 | .04 | -.06 | .02 | .00 | -.00 | .06 | .12 | -.03 | .07 | .12 | -.00 | .09 | .09 | .07 | .27 | .35 | .28 | .24 | .18 | .05 | .21 | -.06 | .22 | .10 | .06 | .16 | .13 | .18 | .49 | — |  |  |
| 39. Bedtime rules school days T1 | .22 | .07 | -.00 | -.14 | .06 | .02 | .22 | .14 | -.01 | -.12 | .05 | -.01 | .07 | .07 | .11 | -.05 | -.01 | .14 | .10 | .06 | .07 | .07 | .13 | .20 | .55 | .43 | .06 | .06 | .04 | -.04 | .04 | .19 | .11 | .11 | .04 | .07 | .28 | .37 | — |  |
| 40. Bedtime rules free days T1 | .15 | .10 | .03 | -.12 | .06 | .06 | .20 | .19 | -.00 | -.12 | .03 | .04 | .04 | .04 | .14 | -.05 | -.02 | .15 | .05 | .07 | .07 | .09 | .17 | .21 | .38 | .55 | .09 | .08 | .13 | -.08 | .09 | .17 | .09 | .11 | .05 | .12 | .22 | .30 | .58 | — |
