# Supplementary material for: Psychosocial Determinants of Sleep Behavior and Healthy Sleep Among Adolescents: A Two-Wave Panel Study
Source: J Youth Adolesc. 2023 Sep 25;53(2):360–73. doi: 10.1007/s10964-023-01866-8 (PMC10764366; doi:10.1007/s10964-023-01866-8)
Supplement: Supplementary file 4 — Appendix 4 [file 10964_2023_1866_MOESM4_ESM.docx]

**Sensitivity analyses: Linear models run without imputation**

**Table 4.1**

*Results of non-imputed linear models: Associations of changes in psychosocial determinants with changes in sleep duration.*

| Sleep Duration | | | | | | |
| --- | --- | --- | --- | --- | --- | --- |
|  | Sleep duration on schooldays | | | Sleep duration on free days | | |
| Explained variance R² | 33.44% | | | 28.11% | | |
| **Determinant** | B | ß | p | B | ß | p |
| Knowledge | -.10 | -.06 | .30 | -.01 | -.01 | .93 |
| Norm-knowledge | .13 | .09 | .04 | -.08 | -.03 | .37 |
| Attitudes | .20 | .18 | <.001 | .06 | .04 | .41 |
| Perceived advantages | .08 | .09 | .06 | .22 | .17 | .001 |
| Self-efficacy | .05 | .05 | .28 | .04 | .03 | .56 |
| Modelling peers | .10 | .10 | .01 | -.00 | -.00 | .97 |
| Perceived norm peers | -.08 | -.09 | .05 | .05 | .04 | .40 |
| Modelling parents | -.00 | -.00 | .92 | .04 | .03 | .52 |
| Perceived norm parents | -.04 | -.04 | .39 | .04 | .03 | .53 |
| Perceived norm parents, related to adolescent behaviour | -.04 | -.04 | .32 | 0.05 | .03 | .49 |
| Perceived barriers | -.07 | -.05 | .25 | -.08 | -.04 | .37 |
| Perceived parental support (encouragement) | -.03 | -.04 | .37 | -.04 | -.04 | .39 |
| Perceived parental support (bedtime rules school days) | .14 | .20 | <.001 |  |  |  |
| Perceived parental support (bedtime rules free days) |  |  | - | .23 | .19 | <.001 |

**Table 4.2**

*Results of non-imputed linear models: Associations of changes in psychosocial determinants with changes in sleep quality (general sleep quality and daytime sleepiness)*

|  | General sleep quality | | | Daytime sleepiness | | |
| --- | --- | --- | --- | --- | --- | --- |
| Explained variance R² | 47.65% | | | 29.35% | | |
| **Determinant** | B | ß | p | B | ß | p |
| Knowledge | -.28 | -.03 | .52 | .76 | .08 | .14 |
| Norm-knowledge | -.29 | -.04 | .30 | -.57 | -.08 | .08 |
| Attitudes | .47 | .08 | .02 | .44 | .08 | .06 |
| Perceived advantages | .31 | .07 | .12 | .08 | .02 | .74 |
| Self-efficacy | .66 | .12 | .001 | -.78 | -.14 | .001 |
| Modelling peers | .10 | .02 | .59 | -.37 | -.07 | .09 |
| Perceived norm peers | -.14 | -.03 | .44 | .12 | .03 | .55 |
| Modelling parents | .28 | .05 | .15 | -.53 | -.09 | .02 |
| Perceived norm parents | -.14 | -.02 | .51 | 021 | .04 | .38 |
| Perceived norm parents, related to adolescent behaviour | .24 | .05 | .22 | -.37 | -.08 | .11 |
| Perceived barriers | -2.63 | -.35 | < .001 | 1.48 | .20 | <.001 |
| Perceived parental support (encouragement) | -.13 | -.03 | .38 | -.07 | -.02 | .71 |
| Perceived parental support (bedtime rules school days) | -.14 | -.04 | .26 | -.06 | -.02 | .69 |
| Perceived parental support (bedtime rules free days) | -.14 | -.031 | .38 | -.06 | -.02 | .72 |

**Table 4.3**

*Results of non-imputed linear models: Associations of changes in psychosocial determinants with changes in sleep quality (sleep onset latencies (SOL) on school days and free days)*

|  | SOL school days | | | SOL free days | | |
| --- | --- | --- | --- | --- | --- | --- |
| Explained variance R² | 33.89% | | | 34.72% | | |
| **Determinant** | B | ß | p | B | ß | p |
| Knowledge | .04 | .06 | .26 | .03 | .04 | .45 |
| Norm-knowledge | -.03 | -.04 | .29 | -.00 | -.01 | .86 |
| Attitudes | -.01 | .03 | .49 | -.01 | -.02 | .64 |
| Perceived advantages | .01 | .03 | .61 | .01 | .03 | .54 |
| Self-efficacy | -.01 | -.01 | .77 | .03 | .06 | .16 |
| Modelling peers | -.01 | -.03 | .46 | -.01 | -.03 | .39 |
| Perceived norm peers | .00 | .01 | .91 | -.01 | -.03 | .52 |
| Modelling parents | -.01 | -.02 | .58 | -.02 | -.03 | .37 |
| Perceived norm parents | .03 | .06 | .14 | .03 | .07 | .07 |
| Perceived norm parents, related to adolescent behaviour | .01 | .03 | .43 | -.00 | -.00 | .99 |
| Perceived barriers | .11 | .19 | <.001 | .09 | .14 | <.001 |
| Perceived parental support (encouragement) | -.01 | -.02 | .68 | -.02 | -.05 | .24 |
| Perceived parental support (bedtime rules school days) | -.00 | -.01 | .87 | - | - | - |
| Perceived parental support (bedtime rules free days) | - | - | - | .01 | .03 | 0.40 |

**Sensitivity analyses: Mixed linear models**

**Table 4.4**

*Results of mixed linear models: Associations of changes in psychosocial determinants with changes in sleep quality (general sleep quality and daytime sleepiness)*

|  | General sleep quality | | | | | Daytime sleepiness | | | | |
| --- | --- | --- | --- | --- | --- | --- | --- | --- | --- | --- |
| Explained variance R² (marginal) | 46.75% | | | | | 28.58% | | | | |
| Explained variance R² (conditional) | 49.00% | | | | | 29.26% | | | | |
| ICC | .016 | | | | | .010 | | | | |
| **Determinant** | B | ß | 95% C.I. | | p | B | ß | 95% C.I. | | p |
|  |  |  | LL | UL |  |  |  | LL | UL |  |
| Knowledge | -.25 | -.02 | -1.11 | .62 | .57 | .76 | .06 | -.24 | 1.76 | .13 |
| Norm-knowledge | -.31 | -.01 | -.86 | .24 | .26 | -.56 | -.01 | -1.19 | .06 | .07 |
| Attitudes | .45 | .07 | .05 | .85 | .03 | .45 | -.01 | -.01 | .90 | .056 |
| Perceived advantages | .32 | .05 | -.07 | .71 | .11 | .07 | -.02 | -.38 | **.52** | .76 |
| Self-efficacy | .68 | .07 | .27 | 1.08 | .001 | -.78 | -.03 | -1.25 | .31 | <.001 |
| Modelling peers | .09 | .01 | .28 | **.**46 | .63 | -.39 | -.03 | -.81 | .03 | .07 |
| Perceived norm peers | -.17 | .01 | -.54 | .19 | .34 | .15 | -.01 | -.27 | .56 | .49 |
| Modelling parents | .30 | .04 | -.08 | .68 | .13 | -.51 | -.03 | -.95 | .07 | .02 |
| Perceived norm parents | -.12 | .01 | -.53 | .28 | .55 | .19 | .00 | -.28 | .67 | .42 |
| Perceived norm parents, related to adolescent behaviour | .23 | .01 | -.16 | .62 | .24 | -.36 | -.04 | -.81 | .08 | .11 |
| Perceived barriers | -2.62 | -.18 | 3.13 | -2.10 | <.001 | 1.47 | .11 | .89 | 2.05 | <.001 |
| Perceived parental support (encouragement) | -.15 | -.01 | -.45 | .16 | .34 | -.06 | .02 | -.42 | .29 | .72 |
| Perceived parental support (bedtime rules school days) | -.13 | -.02 | -.15 | .41 | .37 | -.06 | -.01 | -.34 | .23 | .68 |
| Perceived parental support (bedtime rules free days) | -.14 | -.00 | .44 | .15 | .35 | -.07 | -.04 | -.42 | **.27** | .68 |

**Table 4.5**

*Results of mixed linear models: Associations of changes in psychosocial determinants with changes in sleep quality (sleep onset latencies (SOL) on school days and free days)*

|  | SOL school days | | | | | SOL free days | | | | |
| --- | --- | --- | --- | --- | --- | --- | --- | --- | --- | --- |
| Explained variance R² (marginal) | 34.66% | | | | | 35.52% | | | | |
| Explained variance R² (conditional) | 37.06% | | | | | 38.51 % | | | | |
| ICC | 0.007 | | | | | NA (singular fit), but ANOVA significant | | | | |
| **Determinant** | B | ß | 95% C.I. | | p | B | ß | 95% C.I. | | p |
|  |  |  | LL | UL |  |  |  | LL | UL |  |
| Knowledge | .04 | .05 | -.03 | .11 | .28 | .03 | .03 | -.04 | .11 | .42 |
| Norm-knowledge | -.02 | -.03 | -.07 | .02 | .36 | -.00 | -.03 | -.05 | .05 | .96 |
| Attitudes | -.01 | -.01 | -.04 | .02 | .57 | -.00 | -.01 | -.04 | .03 | .76 |
| Perceived advantages | .01 | -.01 | -.02 | .04 | .57 | .01 | .01 | -.02 | **.05** | .54 |
| Self-efficacy | -.01 | -.03 | -.05 | .02 | .55 | .02 | .01 | -.01 | .06 | .21 |
| Modelling peers | -.00 | .02 | -.04 | .03 | .77 | -.00 | -.00 | -.04 | .03 | .79 |
| Perceived norm peers | -.00 | .02 | -.04 | .02 | .67 | -.02 | -.00 | -.05 | .01 | .30 |
| Modelling parents | -.01 | .01 | -.05 | .02 | .45 | -.02 | -.00 | -.05 | .01 | .25 |
| Perceived norm parents | .03 | .02 | -.01 | .06 | .10 | .04 | .01 | 0 | .07 | .05 |
| Perceived norm parents, related to adolescent behaviour | .02 | .01 | -.02 | .05 | .35 | .00 | -.00 | -.03 | .03 | .98 |
| Perceived barriers | .11 | .10 | .07 | .15 | <.001 | .09 | .08 | .04 | .13 | <.001 |
| Perceived parental support (encouragement) | -.00 | -.00 | -.03 | .02 | .82 | -.01 | -.00 | -.03 | .01 | .32 |
| Perceived parental support (bedtime rules school days) | -.00 | -.00 | -.02 | .02 | .91 | - | - | - | - | - |
| Perceived parental support (bedtime rules free days) | - | - | - | - | - | .02 | .02 | 0 | .04 | .14 |
